# Genetic characterization of the ABO blood group in Neandertals

**DOI:** 10.1186/1471-2148-8-342

**Published:** 2008-12-24

**Authors:** Carles Lalueza-Fox, Elena Gigli, Marco de la Rasilla, Javier Fortea, Antonio Rosas, Jaume Bertranpetit, Johannes Krause

**Affiliations:** 1 Institut de Biologia Evolutiva, UPF-CSIC, Dr. Aiguader 88, 08003 Barcelona, Spain; 2 Área de Prehistoria, Departamento de Historia, Universidad de Oviedo, Teniente Alfonso Martínez s/n, 33011 Oviedo, Spain; 3 Departamento de Paleobiología, Museo Nacional de Ciencias Naturales, CSIC, José Gutierrez Abascal 2, 28006 Madrid, Spain; 4 Department of Evolutionary Genetics, Max Planck Institute for Evolutionary Anthropology, Deutscher Platz 6, 04103 Leipzig, Germany

## Abstract

**Background:**

The high polymorphism rate in the human ABO blood group gene seems to be related to susceptibility to different pathogens. It has been estimated that all genetic variation underlying the human ABO alleles appeared along the human lineage, after the divergence from the chimpanzee lineage. A paleogenetic analysis of the ABO blood group gene in Neandertals allows us to directly test for the presence of the ABO alleles in these extinct humans.

**Results:**

We have analysed two male Neandertals that were retrieved under controlled conditions at the El Sidron site in Asturias (Spain) and that appeared to be almost free of modern human DNA contamination. We find a human specific diagnostic deletion for blood group O (O01 haplotype) in both Neandertal individuals.

**Conclusion:**

These results suggest that the genetic change responsible for the O blood group in humans predates the human and Neandertal divergence. A potential selective event associated with the emergence of the O allele may have therefore occurred after humans separated from their common ancestor with chimpanzees and before the human-Neandertal population divergence.

## Background

The ABO blood group system was the first genetic polymorphism discovered in humans. It consists of three alleles: two co-dominant A and B alleles, and one silent and recessive O allele[1]. The system is controlled by a single gene at the ABO locus. This gene encodes a glycosyltransferase enzyme that adds a sugar residue to a carbohydrate structure known as the H antigen, that is present in the membrane of red cells as well as most epithelial and endothelial cells. The A allele codes for an enzyme that adds a N-acetyl galactosamine to the H antigen, while the B allele, which differs from the former by four amino acid changes, codes for an enzyme that adds a D-galactose. The O allele occurs most frequently in modern humans and carries a human-specific inactivating mutation which produces a non-functional enzyme, and thus the H antigen remains without further modification on the surface of the cells [2,3]. The combination of the three ABO alleles results in four major phenotypes, named A, B, AB and O, these carry different antigens (A, B, both and neither, respectively) that react with specific antibodies [1].

The ABO gene is organized in 7 exons that range in size from 28 to 688 nucleotides, with exon 6 and 7 being the largest [2,4]. Further analyses of the ABO gene have shown that, at the nucleotide sequence level, numerous haplotypes exist among modern humans[5]. The main A and B alleles (hereafter A101 and B101, respectively) differ by four amino acid changes at positions 176, 235, 266 and 268 (nucleotide positions are numbered starting from the first nucleotide of exon 1), with the two later being determining the A and B specificity of the enzyme. The most frequent among all human ABO alleles is the O allele [6]. The most frequent O haplotypes, O01 and O02, share a single G nucleotide deletion in position 261 of exon 6 (Δ261) that creates a premature stop codon and results in a truncated protein with no enzymatic activity [7]; they are referred as the O (Δ261) alleles below. The O01 haplotypes differ from the O02 haplotypes by at least six nucleotide substitutions, including position 297 in exon 6, which is A in the former and G in the latter.

Many studies have examined the association between the ABO alleles and a variety of diseases [8], mainly focussed on infectious agents that can use the A and B antigens as receptors, or in case of the O allele, the absence of the A and B antigens. The O allele can provide a selective advantage since it also produces both anti-A and anti-B antibodies [9]. In particular, it has been suggested that the O allele protects against severe malaria [10]. At the same time, it can be more sensitive to *Helicobacter pylori *infections [11] and to severe forms of cholera [12]. The complex pattern of putative selective agents favouring or acting against different alleles could explain the maintenance of the high ABO polymorphism [13], as evidenced by the signal of balancing selection detected on the gene [14,15]. Therefore studying the evolution of the ABO blood groups to determine when during human history the different alleles emerged may contribute to our understanding on what selective forces might have worked on the different alleles.

Within the apes a convergent evolution of the different blood groups can be observed, although no ape displays all three phenotypes known in humans [3]. Whereas all gorillas studied to date carry only an allele that has properties similar to the human B allele, our closest living relative, the chimpanzee carries two alleles corresponding to the human A and O. Both alleles in the chimpanzee are characterized by species specific mutations in the ABO gene that are distinct from those found in humans [3]. For the ABO alleles on the human lineage it has been estimated that the B101 allele, which gave rise to all the B variants, derived from the ancestral A101 allele around 3.5 million years ago (Mya) [14], whereas the youngest and most common human allele O01, carrying the Δ261 deletion, derived from the A allele only about 1.15 ± 0.2 Mya determined by coalescence analysis [15]. The emergence of the ABO allele O01 therefore predates the mean divergence time for modern humans and Neandertals estimated to more than 500 Kya [16,17] and opens the possibility to put a lower limit on the estimated coalescence date by directly testing for the presence of the O01 allele in the Neandertal population. Thus we undertook a targeted approach to determine whether the Δ261 deletion in the O allele was also present in Neandertals and by that testing the emergence of the O01 allele in the common ancestor of modern human and Neandertal.

## Methods

### DNA extraction and amplification

We analysed two Neandertal samples from the El Sidrón site (Asturias, Spain) [18-20] that were extracted in 2006 under controlled conditions (with excavators completely outfitted in sterile lab gear, including coveralls, face mask, face shield and gloves)[21] and immediately frozen, prior to sending them to the dedicated ancient DNA laboratories in both Barcelona and Leipzig, where DNA extractions were performed. About 500 mg of cortical bone was removed, powdered and extracted as described [20,22].

Analysis of specific nuclear DNA loci from ancient Neandertal remains by the polymerase chain reaction (PCR) is a challenging task that presents two main limitations: the small quantity and short length of ancient DNA fragments due to the degradation of the original template molecules and the difficulty in distinguishing between endogenous Neandertal DNA and putative contemporary modern human contaminants. However, the presence of single informative substitutions in the ABO gene allows for characterization of the different alleles even when retrieving short DNA sequences. Also, the co-amplification of mitochondrial DNA (mtDNA) and nuclear markers allow us to control for the presence of human contamination in the extracts.

Two-step multiplex PCRs [23] in a total volume of 20 μl were set up and incubated for 10 minutes at room temperature with 0.5 units of Shrimp Nuclease (Biotec Pharmacon^®^) to degrade any double-stranded DNA that may contaminate the reagents [24]. After inactivating the nuclease for 30 minutes at 65°C, 5 μl of extract and TaqGold DNA polymerase were added to the PCR. Twenty-seven cycles of PCR were performed in the first PCR and 33 cycles in the second PCR. Reaction conditions were as described [24] except for the annealing temperature, which was 53–55°C, depending on the primers used.

The informative Δ261 position in exon 6 was amplified with the F20144-AGGAAGGATGTCCTCGTGG, R20163-TGCCCTCCCAGACAATGG primer couple (numbered according to the GenBank RefSeq AY268591). The 297 position in exon 6, further retrieved to determine to which of the two main O haplotypes (O01 or O02) the specimens belonged, was amplified with the F20173-TTGGCTGGCTCCCATTGTCTGG, R20194-GAACTGCTCGTTGAGGATG primer couple. The PCR products were 55 and 61 bp in length, respectively. In Barcelona, amplification products of the correct size were cloned using the TOPO TA cloning kit (Invitrogen), and several clones from each product were sequenced using an ABI3730 capillary sequencer (Applied Biosystems) following the manufacturer's instructions. In Leipzig, the products from each PCR were pooled and ligated with a specific tagging sequence for each PCR as described [25] and subsequently sequenced on a 454 FLX machine.

### Contamination controls

To determine the degree of modern human contamination in the Neandertal DNA extracts we amplified and sequenced, using conserved primers, one position in the mitochondrial DNA (mtDNA) coding region (diagnostic transversion 6,267) from each Neandertal extract where Neandertals were found to be distinct from modern humans [17], as well as two diagnostic mtDNA Hypervariable Region I (HVR1) fragments between positions 16,135–16,169 and 16,182–16,223. To ensure that the copy number of nuclear DNA is sufficient for amplifying nuclear Neandertal DNA and to monitor potential nuclear DNA contamination we additionally amplified a control position on the Y-chromosome, which was previously described as ancestral in both specimens and defines the deepest clade in the human Y-chromosome tree (Y2 in [24]). This clade is comprised of all modern human Y chromosomes haplogroups outside Africa. Successful amplifications of the Y chromosomal control fragment confirmed that the two specimens labeled 1253 and 1351c come from two different male individuals [24].

## Results and discussion

In Barcelona, position 261 on exon 6 in the 1253 specimen was amplified in four independent PCRs in parallel with the mtDNA controls (diagnostic transversion 6,267 and mtDNA HVR1 fragments) and the nuclear Y-chromosome position. The products were cloned and at least 10 clones sequenced. The sequences from all four products for the 1253 specimen (n = 53) showed the Δ261 deletion, which defines the O alleles. All Y2 sequences (n = 8) were found to be ancestral (i.e., identical to the chimpanzee sequence), while 35 out of 36 mtDNA sequences were Neandertal-specific. We further amplified and sequenced position 297 on exon 6. Twelve sequences display an A at position 297, which is compatible with the most frequent O haplotype, the O01, but not with the other, the O02 [5]. The position 261 on exon 6 was also amplified in four independent PCRs from the specimen 1351c along with the Y2 fragment and the informative mtDNA position 6,267. All the ABO sequences (n = 55) were identical to those found in the 1253 specimen, while all Y2 sequences (n = 4) were ancestral, and 35 out of 36 mtDNA sequences were Neandertal specific (Table 1).

**Table 1:** Sequencing results for the 1253 and 1351c Neandertal specimens, with ABO polymorphic positions.

Sample	PCR	Δ261	261G	297A	297G	Y2 An	6,267 Ne	6,267 MH	HVR1 Ne	HVR1 MH
1253	1B	15	0	12	0	8	-	-	-	-
1253	2B	10	0	-	-	-	15	1	9	0
1253	3B	15	0	-	-	-	-	-	12	0
1253	4B	13	0	-	-	-	-	-	-	-
1253	1L	39	21	5	0	-	188	29	-	-
1253	2L	40	0	49	21	-	146	32	-	-
1351c	1B	10	0	Na	Na	4	-	-	-	-
1351c	2B	10	0	-	-	-	13	1	-	-
1351c	3B	16	0	-	-	-	-	-	-	-
1351c	4B	19	0	-	-	-	-	-	-	-
1351c	1L	Na	Na	50	0	-	191	31	-	-
1351c	2L	43	41	41	0	-	186	26	-	-

(Δ261): deletion in position 261. (261G): G in position 261. (297A): A in position 297. (297G): G in position 297. (Y2): Y chromosome basal marker. (An): ancestral. (nt 6,267): mtDNA nucleotide position 6,267. (Ne): Neandertal. (MH): Modern human. (HVR1): mtDNA hypervariable region 1. (B): Barcelona; (L): Leipzig. (Na): no amplification product obtained. (-): no amplification attempted.

In Leipzig, position 261 and position 297 on exon 6 in specimen 1253 were amplified in two independent 2-step Multiplex PCRs together with one of the informative mtDNA fragments (position 6,267). All sequences for the 261 fragment in the first PCR show Δ261 (n = 40), for 20 of the sequences we find a additional C > T substitution at position 263, a substitution at that position was not found in any other PCR product from Leipzig or Barcelona and is likely to derive from a miscoding lesion caused by deamination of cytosine on the ancient molecule [26,27]. For the second PCR from specimen 1253, 39 sequences were found to carry the Δ261 and 21 displayed a G at that position (Table 1). At position 297 an A was found in all clones from the first PCR, while in the second PCR most of the sequences (n = 49) display an A and others (n = 21) a G (Table 1). For specimen 1351c, just one PCR resulted in a product covering position 261 where some sequences displayed the Δ261 (n = 43) and some showed no deletion (n = 41). All the sequences (n = 50 and 41) in the two independent PCRs for specimen 1351c showed an A at position 297.

Between 82% and 85% Neandertal sequences were found for the informative mtDNA fragments that were co-amplified along the ABO positions. Despite the informative transversion at position 6,267 where humans have a A and Neandertals a C, there is a second diagnostic position, 6,261, present in that fragment where most humans have a G and Neandertals an A [17]. All clones for the mtDNA fragments show either the 6,267A-6,261G or the 6,267C-6,261A haplotype, allowing to determine whether they derived from human or Neandertal, respectively. There is also a number of transitions present in a minority of the mtDNA sequences that are almost all either C > T or G > A substitutions typical for miscoding lesions [26,27]. Furthermore we find a number of insertions and deletions close to homopolymers, a known problem of the 454 technology [17]. Only little post-mortem damage was found in the nuclear sequences retrieved in Leipzig and Barcelona (see Additional File 1, Table S1), which can be attributed to the small number of individual clone sequences obtained, the short length of the amplified product and a low molecule starting number for the PCR. The later explanation is supported by some unsuccessful amplifications in both Barcelona and Leipzig, and also by the almost equal ratio of clones that show the C > T transition at position 263 for one of the 261 fragments, suggesting the PCR started only from two initial DNA molecules (see Additional File 1 Table S1).

The low level of modern human mtDNA contamination and the fact that from both specimens ancestral Y chromosomal positions could be repeatedly retrieved suggest that the majority of the ABO fragments amplified in this study derive from Neandertal nuclear DNA. Since nine out of eleven successful amplifications of position 261 from both Neandertals only displayed the informative deletion for allele O, we conclude that this allele was present in the Neandertal population. Since four out of five amplifications of position 297 only displayed an A at that position, it is likely that the haplotype was O01. If we assume that our amplifications just start from single molecules and that our Neandertals are heterozygous at position 261, it seems quite unlikely to observe only one of six and one of five PCRs for the second individual without the Δ261 (1253 p = 0.093; 1351c p = 0.15). Given an average contamination rate of 9.7% with a probability of carrying either the A or the B allele of 0.7 (estimated from the current European population) the chances of this observation would increase (1253 p = 0.29; 1351c p = 0.25).

## Conclusion

Our results indicate that the two El Sidrón Neandertal individuals were most likely homozygous for the O01 allele. Nevertheless given a low rate of potential modern human contaminants in an unknown allelic state, we cannot discard the possibility that both Neandertals could have been heterozygous (e.g. OA or OB). The results however suggest the presence of the human O01 allele already in the common ancestor of Neandertals and modern humans and thereby confirming an emergence of the O01 allele more than 1 Mya predating the divergence of the modern human and Neandertal populations. An alternative possibility would be that Neandertals have acquired this allele by gene flow from modern humans, although at present, other genetic evidences suggest that this scenario is unlikely [17,24]. Furthermore a potentially homozygous state in at least two Neandertals, even though from the same group, at least indicates that a potential selective advantage of the O allele could have also been present in the Neandertal population. Future analyses regarding the frequency of the ABO alleles will be necessary to reveal if all three genotypes were conserved in the Neandertal population.

## Authors' contributions

MR, JF and AR excavated the samples and provided paleontological information; CL-F, EG and JK extracted, amplified, sequenced and analyzed ancient DNA data; JB assisted in the interpretation of the results; CL-F and JK wrote the paper.

## Supplementary Material

Additional file 1 **Clone sequences.** Clone sequences generated for the ABO blood group gene and mtDNA fragments in Neandertals. Click here for file
