# Supplementary material for: Genetic characterization of the ABO blood group in Neandertals
Source: BMC Evol Biol. 2008 Dec 24;8:342. doi: 10.1186/1471-2148-8-342 (PMC2629777; doi:10.1186/1471-2148-8-342)
Supplement: Additional file 1 — Clone sequences. Clone sequences generated for the ABO blood group gene and mtDNA fragments in Neandertals. [file 1471-2148-8-342-S1.doc]

**Barcelona:**

Sidrón 1253 PCR1

EXON 6; 261; F20144 R20163

AGGAAGGATGTCCTCGTGGTGACCCCTTGGCTGGCTCCCATTGTCTGGGAGGGCA

B.1.1 . ................

B.1.2 . ................

B.1.3 . ................

B.1.4 . ................

B.1.5 . ................

B.1.6 . ................

B.1.7 . ................

B.1.8 . ................

B.1.9 . ................

B.1.10 . ................

B.1.11 . ................

B.1.12 . ................

B.1.13 . ................

B.1.14 . ................

B.1.15 . ................

Sidrón 1253 PCR1

EXON 6; 297; F20173 R20194

TTGGCTGGCTCCCATTGTCTGGGAGGGCACATTCAACATCGACATCCTCAACGAGCAGTTC

B.1.1 ....................

B.1.2 ....................

B.1.3 ....................

B.1.4 ....................

B.1.5 ....................

B.1.6 ....................

B.1.7 ....................

B.1.8 ....................

B.1.9 ....................

B.1.10 ....................

B.1.11 ....................

B.1.12 ....................

Sidron 1253 PCR2

EXON 6; 261; F20144 R20163

AGGAAGGATGTCCTCGTGGTGACCCCTTGGCTGGCTCCCATTGTCTGGGAGGGCA

B.2.1 . ................

B.2.2 . ................

B.2.3 . ................

B.2.4 . ................

B.2.5 . ................

B.2.6 . ................

B.2.7 . ................

B.2.8 . ................

B.2.9 . ................

B.2.10 . ................

Sidron 1253 PCR3

EXON 6; 261; F20144 R20163

AGGAAGGATGTCCTCGTGGTGACCCCTTGGCTGGCTCCCATTGTCTGGGAGGGCA

B.3.1 . ................

B.3.2 . ................

B.3.3 . ................

B.3.4 . ................

B.3.5 . ................

B.3.6 . ................

B.3.7 . ................

B.3.8 . ................

B.3.9 . ................

B.3.10 . ................

B.3.11 . ................

B.3.12 . ................

B.3.13 . ................

B.3.14 . ................

B.3.15 . ................

Sidron 1253 PCR4

EXON 6; 261; F20144 R20163

AGGAAGGATGTCCTCGTGGTGACCCCTTGGCTGGCTCCCATTGTCTGGGAGGGCA

B.4.1 . ................

B.4.2 . ................

B.4.3 . ................

B.4.4 . ................

B.4.5 . ................

B.4.6 . ................

B.4.7 . ................

B.4.8 . ................

B.4.9 . ................

B.4.10 . ................

B.4.11 . ................

B.4.12 . ................

B.4.13 . ................

Sidrón 1351c PCR1

EXON 6; 261; F20144 R20163

AGGAAGGATGTCCTCGTGGTGACCCCTTGGCTGGCTCCCATTGTCTGGGAGGGCA

B.1.1 . ................

B.1.2 . ................

B.1.3 . ................

B.1.4 . ................

B.1.5 . ................

B.1.6 . ................

B.1.7 . ................

B.1.8 . ................

B.1.9 . ................

B.1.10 . ................

Sidrón 1351c PCR2

EXON 6; 261; F20144 R20163

AGGAAGGATGTCCTCGTGGTGACCCCTTGGCTGGCTCCCATTGTCTGGGAGGGCA

B.2.1 . ................

B.2.2 . ................

B.2.3 . ................

B.2.4 . ................

B.2.5 . ................

B.2.6 . ................

B.2.7 . ................

B.2.8 . ................

B.2.9 . ................

B.2.10 . ................

Sidrón 1351c PCR3

EXON 6; 261; F20144 R20163

AGGAAGGATGTCCTCGTGGTGACCCCTTGGCTGGCTCCCATTGTCTGGGAGGGCA

B.3.1 . ................

B.3.2 . ................

B.3.3 . ................

B.3.4 . ................

B.3.5 . ................

B.3.6 . ................

B.3.7 . ................

B.3.8 . ................

B.3.9 . ................

B.3.10 . ................

B.3.11 . ................

B.3.12 . ................

B.3.13 . ................

B.3.14 . ................

B.3.15 . ................

B.3.16 . ................

Sidrón 1351c PCR4

EXON 6; 261; F20144 R20163

AGGAAGGATGTCCTCGTGGTGACCCCTTGGCTGGCTCCCATTGTCTGGGAGGGCA

B.3.1 . ................

B.3.2 . ................

B.3.3 . ................

B.3.4 . ................

B.3.5 . ................

B.3.6 . ................

B.3.7 . ................

B.3.8 . ................

B.3.9 . ................

B.3.10 . ................

B.3.11 . ................

B.3.12 . ................

B.3.13 . ................

B.3.14 . ................

B.3.15 . ................

B.3.16 . ................

B.3.17 . ................

B.3.18 . ................

B.3.19 . ................

Sidrón 1253

Mitochondrial control (NL16,135-NH16,169)

TGAATATTGTACGGTACCATAAATACTTGACCACCTGTAGTACATAAAAACCCAATCCACATCAAAACCC

B.1.1 ...T........T.....C..............

B.1.2 ...T........T.....C..............

B.1.3 ...T........T.....C..............

B.1.4 ...T........T.....C..............

B.1.5 ...T........T.....C..............

B.1.6 ...T........T.....C..............

B.1.7 ...T........T.....C..............

B.1.8 ...T........T.....C..............

B.1.9 ...T........T.....C..............

B.1.10 ...T........T.....C..............

B.1.11 ...T........T.....C..............

B.1.12 ...T........T.....C..............

Sidrón 1253

Mitochondrial control (NL16,182-NH16,223)

AACCCAATCCACATCAAAACCCCCTCCCC--ATGCTTACAAGCAAGTACAGCAATCAACCCTCAACTATCACACATCAACTGC

B.2.1 C.....C....--...............C.............

B.2.2 C.....C.....................C.............

B.2.3 C.....C....--...............C...N.........

B.2.4 C.....C..----...............C.............

B.2.5 C.....C....--..........N....C.............

B.2.6 C.....C...---...............C.............

B.2.7 C.....C.....................C.............

B.2.8 C.....C....--...............C.............

B.2.9 C.....C....--...............C.............

Sidrón 1253

Y2 control

AACTTAATCAGATTTAGGACACAAAAGCTACTACATAATGAAAAAGAGAGCTGGTGACTT

B.1.1 ......A.........

B.1.2 ......A.........

B.1.3 ......A.........

B.1.4 ......A.........

B.1.5 ......A.........

B.1.6 ......A.........

B.1.7 ......A.........

B.1.8 ......A.........

Sidrón 1253

Mitochondrial control

mtDNA 6,267 TGCTCGCATCTGCTATAGTGGARGCCGGMGCAGGAACAGGTTGAACAGTCTACCCTCCCTTAGCA

B.1.1 ..A.....C................

B.1.2 ..A.....C.............A..

B.1.3 ..A.....C................

B.1.4 ..A.....C................

B.1.5 ..A.....C................

B.1.6 ..A.....C................

B.1.7 ..A.....C................

B.1.8 ..A.....C...AA...........

B.1.9 ..A...T.C................

B.1.10 ..A.....C................

B.1.11 ..A.....C................

B.1.12 ..A.....C................

B.1.13 ..A.....C................

B.1.14 ..A.....C................

B.1.15 ..A.....C................

B.1.16 .........................

Sidrón 1351C

Y2 control

AACTTAATCAGATTTAGGACACAAAAGCTACTACATAATGAAAAAGAGAGCTGGTGACTT

B.1.1 ......A.........

B.1.2 ......A.........

B.1.3 ......A.........

B.1.4 ......A.........

Sidrón 1351C

Mitochondrial control

mtDNA 6,267 TGCTCGCATCTGCTATAGTGGARGCCGGMGCAGGAACAGGTTGAACAGTCTACCCTCCCTTAGCA

B.1.1 ..A.T...C................

B.1.2 ..A.....C................

B.1.3 ..A.....C................

B.1.4 ..A.....C................

B.1.5 ..A.T...C................

B.1.6 ..A.....C................

B.1.7 ..A.....C................

B.1.8 ..A.....C................

B.1.9 ..A.....C................

B.1.10 ..A.....C................

B.1.11 ..A.....C................

B.1.12 A.A.....CA..A...........N

B.1.13 ..A.....C................

B.1.14 .........................

**Leipzig:**

Sidrón 1253 PCR1

Mitochondrial control

mtDNA 6,267 TGCTCGCATCTGCTATAGTGGARGCCGGMGCAGGAACAGGTT GAACAGTCTACCCTCCCTTAGCA

Consensus CV4 13 ..A.....C............. ...

E9MLHLL01AIN1X ...................... ...

E9MLHLL01AJ42A RC ...................... ...

E9MLHLL01AQP4F RC ...................... ...

E9MLHLL01AQDKI ...................... ...

E9MLHLL01ATPKD RC ...................... ...

E9MLHLL01AMEQ7 RC ...................... ...

E9MLHLL01AMOHD ...................... ...

E9MLHLL01AJCCA ...................... ...

E9MLHLL01AL23S RC ...................... ...

E9MLHLL01ANDO9 ...................... ...

E9MLHLL01AOKMB RC ...................... ...

E9MLHLL01ASTUE ...................... ...

E9MLHLL01AR1YW RC ...................... ...

E9MLHLL01AMLNZ ...................... ...

E9MLHLL01AKRPQ ...................... ...

E9MLHLL01AJ1GT RC ...................... ...

E9MLHLL01ARZNS ...................... ...

E9MLHLL01ATDM2 ...................... ...

E9MLHLL01AGZD3 ...................... ...

E9MLHLL01ASUSJ RC ...................... ...

E9MLHLL01AKK13 ...................... ...

E9MLHLL01ASBGS ...................... ...

E9MLHLL01AMR7H ...................... ...

E9MLHLL01AJO68 ...................... ...

E9MLHLL01AS88K ...................... ...

E9MLHLL01AMDLJ RC ...................... ...

E9MLHLL01ATY53 ...................... ...

E9MLHLL01APPCL ...................... ...

E9MLHLL01ASGWH ...................... ...

E9MLHLL01AGJ0V ..A.....C............. ...

E9MLHLL01AKSJ6 RC ..A.....C............. ...

E9MLHLL01APKIL ..A.....C............. ...

E9MLHLL01ARBXT RC ..A.....C............. ...

E9MLHLL01AHDD1 RC ..A.....C............. ...

E9MLHLL01ALH2Q RC ..A.....C............. ...

E9MLHLL01AJZP4 RC ..A.....C............. ...

E9MLHLL01ANGP5 RC ..A.....C............. ...

E9MLHLL01ASUJQ RC ..A.....C............. ...

E9MLHLL01ARXT9 ..A.....C............. ...

E9MLHLL01AGA8Q RC ..A.....C............. ...

E9MLHLL01APYZA ..A.....C............. ...

E9MLHLL01AS0RR RC ..A.....C............. ...

E9MLHLL01AN7ZY RC ..A.....C............. ...

E9MLHLL01AL9IM RC ..A.....C............. ...

E9MLHLL01AP4PW RC ..A.....C............. ...

E9MLHLL01AKLXH RC ..A.....C............. ...

E9MLHLL01AGZLP RC ..A.....C............. ...

E9MLHLL01AOMX0 RC ..A.....C............. ...

E9MLHLL01AGWS3 RC ..A.....C............. ...

E9MLHLL01AL9CO ..A.....C............. ...

E9MLHLL01AQRPC ..A.....C............. ...

E9MLHLL01AMR54 RC ..A.....C............. ...

E9MLHLL01AT8NN RC ..A.....C............. ...

E9MLHLL01AQYD4 RC ..A.....C............. ...

E9MLHLL01AMC39 RC ..A.....C............. ...

E9MLHLL01AHXNT ..A.....C............. ...

E9MLHLL01AQTHF ..A.....C............. ...

E9MLHLL01ASH31 RC ..A.....C............. ...

E9MLHLL01ASW64 ..A.....C............. ...

E9MLHLL01AQ8PV ..A.....C............. ...

E9MLHLL01AMPH1 RC ..A.....C............. ...

E9MLHLL01AQ5UF RC ..A.....C............. ...

E9MLHLL01AP11N RC ..A.....C............. ...

E9MLHLL01ARM9Y RC ..A.....C............. ...

E9MLHLL01AG8V8 RC ..A.....C............. ...

E9MLHLL01AKOL9 RC ..A.....C............. ...

E9MLHLL01AIH94 ..A.....C............. ...

E9MLHLL01AHRGE ..A.....C............. ...

E9MLHLL01ATDKB RC ..A.....C............. ...

E9MLHLL01ATJP6 ..A.....C............. ...

E9MLHLL01ARIK2 ..A.....C............. ...

E9MLHLL01AL697 RC ..A.....C............. ...

E9MLHLL01AGT98 RC ..A.....C............. ...

E9MLHLL01AQ29W RC ..A.....C............. ...

E9MLHLL01AK6UR RC ..A.....C............. ...

E9MLHLL01ATPO4 ..A.....C............. ...

E9MLHLL01ASG34 RC ..A.....C............. ...

E9MLHLL01ALM35 ..A.....C............. ...

E9MLHLL01AK0V4 ..A.....C............. ...

E9MLHLL01AQ8TK RC ..A.....C............. ...

E9MLHLL01ATSS8 RC ..A.....C............. ...

E9MLHLL01ARPI1 RC ..A.....C............. ...

E9MLHLL01AN8A2 ..A.....C............. ...

E9MLHLL01AHF4A RC ..A.....C............. ...

E9MLHLL01AJ710 ..A.....C............. ...

E9MLHLL01AL0A4 ..A.....C............. ...

E9MLHLL01AHLVN ..A.....C............. ...

E9MLHLL01AN59Z RC ..A.....C............. ...

E9MLHLL01APLTQ RC ..A.....C............. ...

E9MLHLL01AP0QP ..A.....C............. ...

E9MLHLL01AL3MB RC ..A.....C............. ...

E9MLHLL01ALW5I ..A.....C............. ...

E9MLHLL01ATPOA RC ..A.....C............. ...

E9MLHLL01ANRKB ..A.....C............. ...

E9MLHLL01APYQD RC ..A.....C............. ...

E9MLHLL01AGXMV RC ..A.....C............. ...

E9MLHLL01AK9W1 RC ..A.....C............. ...

E9MLHLL01AI1J7 RC ..A.....C............. ...

E9MLHLL01AN358 RC ..A.....C............. ...

E9MLHLL01APORN ..A.....C............. ...

E9MLHLL01AHWBG ..A.....C............. ...

E9MLHLL01ARWGL ..A.....C............. ...

E9MLHLL01AG018 ..A.....C............. ...

E9MLHLL01ATI17 ..A.....C............. ...

E9MLHLL01APE9I RC ..A.....C............. ...

E9MLHLL01AGGXC ..A.....C............. ...

E9MLHLL01AQND2 RC ..A.....C............. ...

E9MLHLL01ANOGD RC ..A.....C............. ...

E9MLHLL01AG0Q0 ..A.....C............. ...

E9MLHLL01AKQ2A ..A.....C............. ...

E9MLHLL01AOTID ..A.....C............. ...

E9MLHLL01AIJWG ..A.....C............. ...

E9MLHLL01AK7QK RC ..A.....C............. ...

E9MLHLL01AG481 RC ..A.....C............. ...

E9MLHLL01AFSGY ..A.....C............. ...

E9MLHLL01AQE5A ..A.....C............. ...

E9MLHLL01AP4XW ..A.....C............. ...

E9MLHLL01AGHLQ ..A.....C............. ...

E9MLHLL01AO6PP RC ..A.....C............. ...

E9MLHLL01AODNI RC ..A.....C............. ...

E9MLHLL01AMQL3 ..A.....C............. ...

E9MLHLL01AL260 RC ..A.....C............. ...

E9MLHLL01ASODV RC ..A.....C.......T..... ...

E9MLHLL01AGDX8 ..A.....C............. A..

E9MLHLL01AMK2D ..AA....C............. ...

E9MLHLL01AQTSU ..A.....C....A........ ...

E9MLHLL01AO8XL ..A..T..C............. ...

E9MLHLL01ANMYZ ..A.....C............. ...

E9MLHLL01AQUO8 ..A.....C....A........ ...

E9MLHLL01AIQCX RC ..A.....C...A......... ...

E9MLHLL01AOAB2 ..A.....C.......T..... ...

E9MLHLL01AIS9U RC ..A.....C.T........... ...

E9MLHLL01ATV1T RC ..A.....C.T........... ...

E9MLHLL01ARLXB ..AA....C............. ...

E9MLHLL01AGBDH RC ..A..T..C............. ...

E9MLHLL01ANTS8 RC ..A.....C...A......... ...

E9MLHLL01AMLSO RC ..AA....C............. ...

E9MLHLL01AG0LB ..A.....C.......T..... ...

E9MLHLL01ASHEQ RC ..A.....C.......T..... ...

E9MLHLL01AS9GF RC ..AA....C............. ...

E9MLHLL01AGZKP ..A.....C.......T..... ...

E9MLHLL01AQT8B ..A.....C....A........ ...

E9MLHLL01ASAF7 ..A...A.C............. ...

E9MLHLL01AGKNG ..A.....C............. A..

E9MLHLL01AJKGX ..A.....C...A......... ...

E9MLHLL01AQJQZ RC ..A..T..C............. ...

E9MLHLL01AKNNK RC ..A.....C............. A..

E9MLHLL01AO2WY ..A.....C...A......... ...

E9MLHLL01AR7W6 ..A...A.C............. ...

E9MLHLL01AP5DT ..A.....C......G...... ...

E9MLHLL01ASULP RC ..A.....C......G...... ...

E9MLHLL01AJ6QA RC ..A....AC............. ...

E9MLHLL01AK5P6 RC ..A.....C....A........ ...

E9MLHLL01AIHRN ..A.....C............. ..G

E9MLHLL01AJFRK RC ..A.T...C............. ...

E9MLHLL01APYMV ..A ....C............. ...

E9MLHLL01AGGMT RC ..A.....C............. ..

E9MLHLL01ASQDF ..A ....C............. ...

E9MLHLL01ASL0S ..AA....C............. ...

E9MLHLL01AJ6P7 RC ..A....AC............. ...

E9MLHLL01ARL9C RC ..A.TT..C............. ...

E9MLHLL01AJOSC RC ..A.TT..C............. ...

E9MLHLL01AQ2CJ RC ..A.T...T............. ...

E9MLHLL01AJR6J ..AA....CA............ ...

E9MLHLL01AHLB8 ..AA....CA............ ...

E9MLHLL01ASQG8 ..A.T...T............. ...

E9MLHLL01ALWHP ..A...AAC............. ...

E9MLHLL01AS038 RC ..A.TT..C............. ...

E9MLHLL01AS6YV RC ..A.TT..C............. ...

E9MLHLL01ARG25 RC ..A.....C.........AA.. ...

E9MLHLL01ALQT9 ..A.....C.T.....T..... ...

E9MLHLL01AI2NN ..AA..A.C............. ...

E9MLHLL01AGTBL RC ..A.....C....A....A... ...

E9MLHLL01ANQKS ..A...AAC............. ...

E9MLHLL01AJTQ2 RC ..A...A.C...A......... ...

E9MLHLL01AS3BY ..AA....CA............ ...

E9MLHLL01AGXM1 ..A.TT..C............. ...

E9MLHLL01AJVZL RC A.AA....C............. ...

E9MLHLL01AJTA8 RC ..A.....C.T.....T..... ...

E9MLHLL01ASBEY RC ..A.....T.T........... ...

E9MLHLL01AKPLY A.AA....C............. ...

E9MLHLL01AQZ18 ..A.TT..C............. ...

E9MLHLL01AG3TH ..A.TT..C............. ...

E9MLHLL01AKZBS RC ..AA....CA............ ...

E9MLHLL01AI6NZ ..AA..A.C............. ...

E9MLHLL01ALAO9 RC ..A.....C.........AA.. ...

E9MLHLL01AFUYH RC ..A.....C............. A..

E9MLHLL01AMDSR RC ..A.....C.T.....T..... ...

E9MLHLL01AJCMN RC ..A.TT..C............. ...

E9MLHLL01AMH0E RC ..A...AAC............. ...

E9MLHLL01AL7BS ..A.....C...AA........ ...

E9MLHLL01ARQI5 RC ..A.....CA.G.......... ...

E9MLHLL01AHMOI ..A.....C....A....A... ...

E9MLHLL01AGAZ1 RC ..A.....C............. ...

E9MLHLL01AS20F ..A ....C...A.........T...

E9MLHLL01AI7RW ..A...AAC...A......... ...

E9MLHLL01ANYXU RC ..AA....C..........A.. A..

E9MLHLL01AFY2X RC ..A....ACA..A......... ...

E9MLHLL01ASX8E RC ..A.....C...AA....A... ...

E9MLHLL01AS9XP RC ..A.....C... A....A... ...

E9MLHLL01ALKFG RC ..A..T..T..C.......... ...

E9MLHLL01AHJB1 ..A.....C...A.....A... ...

E9MLHLL01AOYHU RC ..AA..AACA............ ...

E9MLHLL01AHOP8 RC ..AA..AACA............ ...

E9MLHLL01AJX80 ..A....ACA..A......A.. ...

E9MLHLL01APE83 RC ..A..T..T..C.......... .G.

E9MLHLL01ARGNL RC A.AA....C...AA........ ...

E9MLHLL01AKPJU ..A..T..T..C.......... .G.

E9MLHLL01AKZAA ..A.....C.T.... T..... ...

E9MLHLL01AHQJA ..A....ACA..AA........ ...

E9MLHLL01ANEVX RC A.AA..AACA............ ...

E9MLHLL01AOG4P RC A.AA..AACA............ ...

E9MLHLL01AGDGY RC ..A..T..T..C.......... .G.

E9MLHLL01AOBKA RC A.AA..AACA............ ...

E9MLHLL01ARXYD RC ..AA...AC... A....AA.. ...

E9MLHLL01AIGTV ..A.....C...A......A..T...

E9MLHLL01ARPPZ ..A...AACA..AA........ A..

Sidron 1253 PCR1

EXON 6; 261; F20144 R20163

ABO1 10 AGGAAGGATGTCCTCGTGGTG ACCCCTTGGCTGGCTCCCATTGTCTGGGAGGGCAT

Consensus ABO1 10 . ................

E9MLHLL01APHGG .. ................

E9MLHLL01AR6CR .. ................

E9MLHLL01ANI24 .. ................

E9MLHLL01AOTN2 .. ................

E9MLHLL01AR594 .. ................

E9MLHLL01AO6PM .. ................

E9MLHLL01AMIYB .. ................

E9MLHLL01AI3TG RC .. ................

E9MLHLL01AMFNX .. ................

E9MLHLL01AIRS7 .. ................

E9MLHLL01AID2N RC .. ................

E9MLHLL01ALVT1 .. .... ...........

E9MLHLL01ALTRW RC .. ................

E9MLHLL01AOQQX .. ................

E9MLHLL01ARTFF RC .. ................

E9MLHLL01ATPFW .. ................

E9MLHLL01AOI9X .. ................

E9MLHLL01AOYZL .. ................

E9MLHLL01AOZT1 RC .. ................

E9MLHLL01AMODJ RC .. ................

E9MLHLL01AKV6O RC .CG.... ...........

E9MLHLL01ATM1P RC . ................

E9MLHLL01AM2XF . ................

E9MLHLL01AIQ6I RC . ................

E9MLHLL01AG9EW RC . ................

E9MLHLL01ANT72 RC . ................

E9MLHLL01ALV22 . ................

E9MLHLL01AHIIZ RC . ................

E9MLHLL01ATGL0 RC . ................

E9MLHLL01AH5T4 . ................

E9MLHLL01ALY5C . ................

E9MLHLL01AK2KH RC . ................

E9MLHLL01ATCK2 RC . ................

E9MLHLL01ATCNV RC . ................

E9MLHLL01AKYDE RC . ................

E9MLHLL01ASN7E RC . ................

E9MLHLL01AMPEX RC . ................

E9MLHLL01AOJYC . ................

E9MLHLL01AKE5O RC . ................

E9MLHLL01ARQDW RC . ................

E9MLHLL01ARBUD . ................

E9MLHLL01AJTBP RC . ................

E9MLHLL01AQBNB RC . ................

E9MLHLL01AQXOC RC . ................

E9MLHLL01AIO4P . ................

E9MLHLL01AN494 RC . ................

E9MLHLL01AHMDC RC . ................

E9MLHLL01AS3BP RC . ................

E9MLHLL01AN5XF . ................

E9MLHLL01AK056 . .... ...........

E9MLHLL01AGNY4 . .... ...........

E9MLHLL01ARWJD . .... ...........

E9MLHLL01APU64 . .... ...........

E9MLHLL01AJ96N . .... ...........

E9MLHLL01ATM1H . .... ...........

E9MLHLL01AIVF7 . .... ...........

E9MLHLL01AKR1W RC . ...............

E9MLHLL01AHZII . .... ...........

E9MLHLL01AKFPV RC . .... ..........

Sidrón 1253 PCR1

EXON 6; 297; F20173 R20194

ABO2 11 TTGGCTGGCTCCCATTGTCTGGGAGGGCA TTCAACATCGACATCCTCAACGAGCAGTTC

Consensus ABO2 11 .......CA...........

E9MLHLL01AIG57 RC .......CA...........

E9MLHLL01AHK3J RC .......CA...........

E9MLHLL01AHJJJ .......CA...........

E9MLHLL01APMBY RC .......CA...........

E9MLHLL01APF0G RC .......CA...........

Sidrón 1253 PCR2

Mitochondrial control

mtDNA 6,267 TGCTCGCATCTGCTATAGTGGARGCCGGMGCAGGAACAGGTTG AACAGTCTACCCTCCCTTAGCA

Consensus CV4 13 . .A.....C.............. ..

E9MLHLL01AF69A RC . .A.....C.............. ..

E9MLHLL01ART0K RC . .A.....C.............. ..

E9MLHLL01AQRXO RC . .A.....C.............. ..

E9MLHLL01ASWZL RC . ...................... ..

E9MLHLL01AJWBY . ...................... ..

E9MLHLL01AG2JJ RC . ...................... ..

E9MLHLL01AQH4D . ...................... ..

E9MLHLL01AS63M RC . ...................... ..

E9MLHLL01AOGAH RC . ...................... ..

E9MLHLL01AJ4LK . ...................... ..

E9MLHLL01AOD4V . ...................... ..

E9MLHLL01AR2T4 RC . ...................... ..

E9MLHLL01AIZXL . ...................... ..

E9MLHLL01AG9E2 . ...................... ..

E9MLHLL01AH7Y6 RC . ...................... ..

E9MLHLL01AR1IB RC . ...................... ..

E9MLHLL01ARTY4 . ...................... ..

E9MLHLL01AQOFV . ...................... ..

E9MLHLL01ANLTZ . ...................... ..

E9MLHLL01AQ235 . ...................... ..

E9MLHLL01ALBMJ . ...................... ..

E9MLHLL01ALHX1 . ...................... ..

E9MLHLL01AQ8RV . ...................... ..

E9MLHLL01AGGFF . ...................... ..

E9MLHLL01ASKCO . ...................... ..

E9MLHLL01AF1T8 RC . ...................... ..

E9MLHLL01AQ2F9 . ...................... ..

E9MLHLL01ANSC2 RC . ...................... ..

E9MLHLL01APCTE RC . ...................... ..

E9MLHLL01AR4WF RC . ...................... ..

E9MLHLL01ARY2K RC . ...................... ..

E9MLHLL01ARTXL . ...................... ..

E9MLHLL01AH8EP RC ........................ ..

E9MLHLL01AHALL . ...................... ..

E9MLHLL01ANQ9K RC . .....................A ..

E9MLHLL01AIAAQ . .A.................... ..

E9MLHLL01AN6NR RC . .A.....C.............. ..

E9MLHLL01APEGQ . .A.....C.............. ..

E9MLHLL01AJJJI RC . .A.....C.............. ..

E9MLHLL01AIBNT . .A.....C.............. ..

E9MLHLL01AMG3O RC . .A.....C.............. ..

E9MLHLL01AKQZ4 . .A.....C.............. ..

E9MLHLL01ANA4W RC . .A.....C.............. ..

E9MLHLL01AMEO7 . .A.....C.............. ..

E9MLHLL01AQNW0 RC . .A.....C.............. ..

E9MLHLL01ARL8C RC . .A.....C.............. ..

E9MLHLL01AIIFA . .A.....C.............. ..

E9MLHLL01AR5E0 . .A.....C.............. ..

E9MLHLL01APE46 . .A.....C.............. ..

E9MLHLL01AOQPP RC . .A.....C.............. ..

E9MLHLL01AKFUT RC . .A.....C.............. ..

E9MLHLL01AJ7S3 . .A.....C.............. ..

E9MLHLL01AL58L RC . .A.....C.............. ..

E9MLHLL01APECA . .A.....C.............. ..

E9MLHLL01AHXXK . .A.....C.............. ..

E9MLHLL01APU6Q . .A.....C.............. ..

E9MLHLL01AIA8M RC . .A.....C.............. ..

E9MLHLL01AOPI1 RC . .A.....C.............. ..

E9MLHLL01AP40I . .A.....C.............. ..

E9MLHLL01AM7UO . .A.....C.............. ..

E9MLHLL01AOILH . .A.....C.............. ..

E9MLHLL01AMQLX . .A.....C.............. ..

E9MLHLL01AJWGU . .A.....C.............. ..

E9MLHLL01AO4XO RC . .A.....C.............. ..

E9MLHLL01AK0IL RC . .A.....C.............. ..

E9MLHLL01ARC01 RC . .A.....C.............. ..

E9MLHLL01AT15S RC . .A.....C.............. ..

E9MLHLL01ARQNB RC . .A.....C.............. ..

E9MLHLL01ANN00 . .A.....C.............. ..

E9MLHLL01ARPMH RC . .A.....C.............. ..

E9MLHLL01ARIZH RC . .A.....C.............. ..

E9MLHLL01ALTG0 RC . .A.....C.............. ..

E9MLHLL01AFL4J RC . .A.....C.............. ..

E9MLHLL01AHH7D . .A.....C.............. ..

E9MLHLL01AJQF1 . .A.....C.............. ..

E9MLHLL01AHCAM . .A.....C.............. ..

E9MLHLL01AIW8C . .A.....C.............. ..

E9MLHLL01AHO0E RC . .A.....C.............. ..

E9MLHLL01AJ4SU RC . .A.....C.............. ..

E9MLHLL01AIJ6U . .A.....C.............. ..

E9MLHLL01AOBFG . .A.....C.............. ..

E9MLHLL01APXJS RC . .A.....C.............. ..

E9MLHLL01ARYTT . .A.....C.............. ..

E9MLHLL01AOM7F RC . .A.....C.............. ..

E9MLHLL01AQQ6G . .A.....C.............. ..

E9MLHLL01AQNW0 RC . .A.....C.............. ..

E9MLHLL01ARL8C RC . .A.....C.............. ..

E9MLHLL01AIIFA . .A.....C.............. ..

E9MLHLL01AR5E0 . .A.....C.............. ..

E9MLHLL01APE46 . .A.....C.............. ..

E9MLHLL01AOQPP RC . .A.....C.............. ..

E9MLHLL01AKFUT RC . .A.....C.............. ..

E9MLHLL01AJ7S3 . .A.....C.............. ..

E9MLHLL01AL58L RC . .A.....C.............. ..

E9MLHLL01AL4IT . .A.....C.............. ..

E9MLHLL01ATMN7 RC . .A.....C.............. ..

E9MLHLL01AFO78 . .A.....C.............. ..

E9MLHLL01AGOKH . .A.....C.............. ..

E9MLHLL01ASIKF . .A.....C.............. ..

E9MLHLL01ARCMM RC . .A.....C.............. ..

E9MLHLL01AM510 . .A.....C.............. ..

E9MLHLL01AOWTL . .A.....C.............. ..

E9MLHLL01AP5F9 RC . .A.....C.............. ..

E9MLHLL01AJBFC . .A.....C.............. ..

E9MLHLL01AGDF9 RC . .A.....C.............. ..

E9MLHLL01AJ3MD . .A.....C.............. ..

E9MLHLL01APA86 RC . .A.....C.............. ..

E9MLHLL01AJXAV . .A.....C.............. ..

E9MLHLL01AQT8I RC . .A.....C.............. ..

E9MLHLL01AQ25A RC . .A.....C.............. ..

E9MLHLL01AMEN4 . .A.....C.T............ ..

E9MLHLL01AQWZH . .A.....C.............A ..

E9MLHLL01AIWBJ . .A.....C.............A ..

E9MLHLL01AM429 . .A..T..C.............. ..

E9MLHLL01AMP4W RC . .A.....C.T............ ..

E9MLHLL01APXXR . .A.....CA............. ..

E9MLHLL01AJPSG RC . .A.T...C.............. ..

E9MLHLL01ANG85 . .A.....C..........A... ..

E9MLHLL01AJO8J RC . .A....AC.............. ..

E9MLHLL01AO1UH RC . .A.....C....A......... ..

E9MLHLL01AM445 RC . .A...T.C.............. ..

E9MLHLL01AP95J . .A.....C...A.......... ..

E9MLHLL01AJRWZ . .A.....T.............. ..

E9MLHLL01AS6JY . .A.....C.........A.... ..

E9MLHLL01ASBE4 RC . .AA....C.............. ..

E9MLHLL01AKX2C . .A.....C.......T...... ..

E9MLHLL01AIOJI . .A..T..C.............. ..

E9MLHLL01AIKGL RC A .A.....C.............. ..

E9MLHLL01ATJQD RC . .A.....C.T............ ..

E9MLHLL01AQETH RC . .A.....C.......T...... ..

E9MLHLL01AQ6TE RC . .A.....C.........A.... ..

E9MLHLL01AJ12L . .AA....C.............. ..

E9MLHLL01AN0KX . .A.....C...A.......... ..

E9MLHLL01ARWPL RC . .A.....C....A......... ..

E9MLHLL01AMVFA . .A.....CA............. ..

E9MLHLL01ALKPC . .A.....CA............. ..

E9MLHLL01AL0G8 . .A.T...C.............. ..

E9MLHLL01ASRV3 RC . .A.....C.A............ ..

E9MLHLL01AN6WF . .A.....C...A.......... ..

E9MLHLL01AMYRR . .A..T..C.............. ..

E9MLHLL01AIUU0 . .A..T..C.............. ..

E9MLHLL01AN86G . .A.....C...AA......... ..

E9MLHLL01ARLW8 RC . .A.....C...AA......... ..

E9MLHLL01ASXNB RC . .A.....C..........A..A ..

E9MLHLL01AG5L2 . .A.. ..C...... ....... ..

E9MLHLL01AHVN9 . .A.....C.T.....T...... ..

E9MLHLL01AHWI5 RC . .A.....C..........A..A ..

E9MLHLL01APUKU . .A.....CA.........A... ..

E9MLHLL01AJCB4 RC . .A.....C....A.....A... ..

E9MLHLL01ART02 . .A.T...T.............. ..

E9MLHLL01AK1HR RC . .A.TT..C.............. ..

E9MLHLL01AG80Z RC A .AA....C.............. ..

E9MLHLL01AK8LZ RC . .A.....C....A.....A... ..

E9MLHLL01AHJNI A .A.....C..........T... ..

E9MLHLL01AP757 . .A.....C...A.........A ..

E9MLHLL01AL5W8 . .A.....C.........AA... ..

E9MLHLL01AH0WD RC . .A.....C....A.....A... ..

E9MLHLL01APYR0 . .A.....C... A......... ..

E9MLHLL01ARV48 . .A.T...T.............. ..

E9MLHLL01ANTJO RC . .A.....CA..A.......... ..

E9MLHLL01ARGQB RC . .A.....C....A.....A... ..

E9MLHLL01AR8VC RC . .AA..A.C.............. ..

E9MLHLL01AQ0PV RC . .A.TT..C.............. ..

E9MLHLL01ATYR8 . .A.....C...AA......... ..

E9MLHLL01AIQ35 A .AA....CA............. ..

E9MLHLL01AHLHP . .A.....CA.. A......... ..

E9MLHLL01APFP9 RC A .AA....CA............. ..

E9MLHLL01ATGLX RC . .A...AACA...A......... ..

E9MLHLL01ARYNB . .A...AACA...A......... ..

E9MLHLL01AK782 RC . .A.....C...AA....AA..A ..

E9MLHLL01AIMZ2 RC . .A...AACA..AA......... ..

E9MLHLL01AR2F0 RC . .AA..AACA..A.......... ..

E9MLHLL01AR59H . .A...AACA..AA......... ..

E9MLHLL01AI7HQ RC . .AA..AACA..AA......... ..

E9MLHLL01ARMYN RC A .AA....CA.............T..

E9MLHLL01AOJYS RC A .AA..AAC...A......A... ..

E9MLHLL01AJO77 . .AA..AACA.. A....A.... ..

E9MLHLL01AMAWQ RC . .AA..AACA..AA......... ..

Sidron 1253 PCR2

EXON 6; 261; F20144 R20163

ABO1 10 AGGAAGGATGTCCTCGTGGTGACCCCTTGGCTGGCTCCCATTGTCTGGGAGGGCA

Consensus ABO1 10 . ................

E9MLHLL01AN93N RC . ................

E9MLHLL01ASEUG RC . ................

E9MLHLL01AG81P RC . ................

E9MLHLL01ASXBQ . ................

E9MLHLL01ANXV3 . ................

E9MLHLL01AMF5U RC . ................

E9MLHLL01AM16S . ................

E9MLHLL01ARDQI RC . ................

E9MLHLL01AJ540 . ................

E9MLHLL01AMOVZ RC . ................

E9MLHLL01AO6N0 RC . ................

E9MLHLL01AHUP2 . ................

E9MLHLL01ASJ1I . ................

E9MLHLL01APS9M . ................

E9MLHLL01ASURI RC . ................

E9MLHLL01AKNS6 RC . .T..............

E9MLHLL01ATZAF . .... ...........

E9MLHLL01AR4MR . .... ...........

E9MLHLL01ASETF RC . .T..............

E9MLHLL01ATPA9 RC . .T..............

E9MLHLL01AOC6K RC . .T..............

E9MLHLL01ANA7J . .T..............

E9MLHLL01AO2PN RC . .T..............

E9MLHLL01AINKE . .T..............

E9MLHLL01AIZ6L RC . .T..............

E9MLHLL01ATGQD . .T..............

E9MLHLL01ARS8U . .... ...........

E9MLHLL01AR9JY RC . .T..............

E9MLHLL01AJWLF . .T..............

E9MLHLL01ANR7W . .... ...........

E9MLHLL01AKQUY RC . .T..............

E9MLHLL01ANK2R . .T..............

E9MLHLL01AH1LO . .T..............

E9MLHLL01AQ6WV . .... ...........

E9MLHLL01AS0RX . .T..............

E9MLHLL01AMDJY RC . .T..............

E9MLHLL01AIGOG . .T..............

E9MLHLL01AJFQ0 . .T..............

E9MLHLL01AKEUV . .T..............

E9MLHLL01AKRYP RC . .T..............

Sidrón 1253 PCR2

EXON 6; 297; F20173 R20194

ABO2 11 TTGGCTGGCTCCCATTGTCTGGGAGGGCA TTCAACATCGACATCCTCAACGAGCAGTTC

Consensus ABO2 11 .......CA ...........

E9MLHLL01ASNU0 RC .......CG ...........

E9MLHLL01AHLZR .......CG ...........

E9MLHLL01AMQFO .......CG ...........

E9MLHLL01ASE7O RC .......CG ...........

E9MLHLL01AK16O RC .......CG ...........

E9MLHLL01AISCU .......CG ...........

E9MLHLL01AHNE0 RC .......CG ...........

E9MLHLL01AH82P .......CG ...........

E9MLHLL01AM2VD .......CG ...........

E9MLHLL01AQXXT RC .......CG ...........

E9MLHLL01AMFO7 RC .......CG ...........

E9MLHLL01AS6BN RC .......CG ...........

E9MLHLL01AMO13 .......CG ...........

E9MLHLL01AJ5PT .......CG ...........

E9MLHLL01AKYTZ RC .......CG ...........

E9MLHLL01AIT8T RC .......CGG...........

E9MLHLL01AKLLV RC .......CG ...........

E9MLHLL01AQ0VM .......CG ...........

E9MLHLL01AHZNC RC .......CG ...........

E9MLHLL01AQJ1B RC .G.....CG ...........

E9MLHLL01AJ5RU .......CG ...........

E9MLHLL01AIWBP .......CA ...........

E9MLHLL01AMMIZ RC .......CA ...........

E9MLHLL01ALPYT RC .......CA ...........

E9MLHLL01AGTI7 RC .......CA ...........

E9MLHLL01AR8O5 .......CA ...........

E9MLHLL01ARKHH RC .......CA ...........

E9MLHLL01AI9C8 RC .......CA ...........

E9MLHLL01ALTS0 .......CA ...........

E9MLHLL01AJPHP .......CA ...........

E9MLHLL01AJJ4Q .......CA ...........

E9MLHLL01AIZ1A RC .......CA ...........

E9MLHLL01APLPT .......CA ...........

E9MLHLL01AJMUN .......CA ...........

E9MLHLL01ARNKY .......CA ...........

E9MLHLL01AQ2LF .......CA ...........

E9MLHLL01ANM6T RC .......CA ...........

E9MLHLL01AMEWL .......CA ...........

E9MLHLL01ASTH7 RC .......CA ...........

E9MLHLL01AQBL7 RC .......CA ...........

E9MLHLL01AGD4P .......CA ...........

E9MLHLL01AMF1N .......CA ...........

E9MLHLL01AQQH6 .......CA ...........

E9MLHLL01AIIIZ RC .......CA ...........

E9MLHLL01AHK7G RC .......CA ...........

E9MLHLL01AMCR3 RC .......CA ...........

E9MLHLL01AM5J6 .......CA ...........

E9MLHLL01ARU1Q RC .......CA ...........

E9MLHLL01AIRIE .......CA ...........

E9MLHLL01AI0EQ RC .......CA ...........

E9MLHLL01AKZ9Y .......CA ...........

E9MLHLL01AG5II .......CA ...........

E9MLHLL01AQAWV RC .......CA ...........

E9MLHLL01AJ3KC RC .......CA ...........

E9MLHLL01AGJX7 RC .......CA ...........

E9MLHLL01ATABL .......CA ...........

E9MLHLL01ARP3V .......CA ...........

E9MLHLL01AN949 .......CA ...........

E9MLHLL01AJ1JZ RC .......CA ...........

E9MLHLL01ANOAQ .......CA ...........

E9MLHLL01AOM6H .......CA ...........

E9MLHLL01ASFNI .......CA ...........

E9MLHLL01AGHO3 .......CA ...........

E9MLHLL01ARCKR .......CA ...........

E9MLHLL01AR8SU .......CA ...........

E9MLHLL01AJQ6U .......CA ...........

E9MLHLL01AQHXP RC .......CA ...........

E9MLHLL01AJW8U RC .......CA ...........

E9MLHLL01AHLBQ RC A......CA ...........

E9MLHLL01AKHAJ RC .......CA ....G......

Sidrón 1351c PCR1

Mitochondrial control

mtDNA 6,267 TGCTCGCATCTGCTATAGTGGARG CC GG M GC A GGAACAGGTT G AACAGTCTACCCTCCCTTAGCA

Consensus CV4 13 ..A. .. .. C .. . .......... . ..

E9MLHLL01ASUEW .... .. .. . .. . .......... . ..

E9MLHLL01AQR7F .... .. .. . .. . .......... . ..

E9MLHLL01ATF02 RC .... .. .. . .. . .......... . ..

E9MLHLL01AQHDH .... .. .. . .. . .......... . ..

E9MLHLL01ARYAQ RC .... .. .. . .. . .......... . ..

E9MLHLL01ANIVA .... .. .. . .. . .......... . ..

E9MLHLL01AL0UW RC .... .. .. . .. . .......... . ..

E9MLHLL01AQNV5 .... .. .. . .. . .......... . ..

E9MLHLL01AINET RC .... .. .. . .. . .......... . ..

E9MLHLL01AJ12O RC .... .. .. . .. . .......... . ..

E9MLHLL01AKCHQ .... .. .. . .. . .......... . ..

E9MLHLL01AK5QN .... .. .. . .. . .......... . ..

E9MLHLL01AKSUN RC .... .. .. . .. . .......... . ..

E9MLHLL01AHDRO .... .. .. . .. . .......... . ..

E9MLHLL01AGKJY RC .... .. .. . .. . .......... . ..

E9MLHLL01AOWX6 .... .. .. . .. . .......... . ..

E9MLHLL01AGDH1 .... .. .. . .. . .......... . ..

E9MLHLL01ATI24 RC .... .. .. . .. . .......... . ..

E9MLHLL01ANAVD RC .... .. .. . .. . .......... . ..

E9MLHLL01ANIZ7 .... .. .. . .. . .......... . ..

E9MLHLL01AKIF9 .... .. .. . .. . .......... . ..

E9MLHLL01AG18U .... .. .. . .. . .......... . ..

E9MLHLL01AOMZL .... .. .. . .. . .......... . ..

E9MLHLL01ANCSJ RC .... .. .. . .. . .......... . ..

E9MLHLL01AK39V RC .... .. .. . .. . .......... . ..

E9MLHLL01AGUD5 .... .. .. . .. . .......... . ..

E9MLHLL01AH7CB RC .... .. .. . .. . .......... . ..

E9MLHLL01ATCTB .... .. .. . .. . .......... . ..

E9MLHLL01AN10S RC .... .. .. . .. . .......... . ..

E9MLHLL01AP358 .... .. .. . .. . .......... . ..

E9MLHLL01ALT64 .... .. .. . .. . .......... . ..

E9MLHLL01AI5RC RC ..A. .. .. C .. . .......... . ..

E9MLHLL01AQ1FU RC ..A. .. .. C .. . .......... . ..

E9MLHLL01ALSLQ RC ..A. .. .. C .. . .......... . ..

E9MLHLL01AI3PF RC ..A. .. .. C .. . .......... . ..

E9MLHLL01ASWQ2 ..A. .. .. C .. . .......... . ..

E9MLHLL01AJF4X ..A. .. .. C .. . .......... . ..

E9MLHLL01AKKIB RC ..A. .. .. C .. . .......... . ..

E9MLHLL01AGVXW RC ..A. .. .. C .. . .......... . ..

E9MLHLL01AMBJ0 RC ..A. .. .. C .. . .......... . ..

E9MLHLL01AP06J ..A. .. .. C .. . .......... . ..

E9MLHLL01AQ9XD RC ..A. .. .. C .. . .......... . ..

E9MLHLL01AK4TI RC ..A. .. .. C .. . .......... . ..

E9MLHLL01AINEE RC ..A. .. .. C .. . .......... . ..

E9MLHLL01AJLTL RC ..A. .. .. C .. . .......... . ..

E9MLHLL01AKYE0 ..A. .. .. C .. . .......... . ..

E9MLHLL01AJJR8 RC ..A. .. .. C .. . .......... . ..

E9MLHLL01AH1YK ..A. .. .. C .. . .......... . ..

E9MLHLL01ANEQI ..A. .. .. C .. . .......... . ..

E9MLHLL01APBQ5 RC ..A. .. .. C .. . .......... . ..

E9MLHLL01ATMPA ..A. .. .. C .. . .......... . ..

E9MLHLL01AHSDM RC ..A. .. .. C .. . .......... . ..

E9MLHLL01ATGMI ..A. .. .. C .. . .......... . ..

E9MLHLL01AILXF RC ..A. .. .. C .. . .......... . ..

E9MLHLL01AJ2RP RC ..A. .. .. C .. . .......... . ..

E9MLHLL01ANR3M ..A. .. .. C .. . .......... . ..

E9MLHLL01AMQKL ..A. .. .. C .. . .......... . ..

E9MLHLL01AG840 RC ..A. .. .. C .. . .......... . ..

E9MLHLL01AHU61 RC ..A. .. .. C .. . .......... . ..

E9MLHLL01AQZ52 RC ..A. .. .. C .. . .......... . ..

E9MLHLL01AJD59 RC ..A. .. .. C .. . .......... . ..

E9MLHLL01ARTPO ..A. .. .. C .. . .......... . ..

E9MLHLL01AG67A RC ..A. .. .. C .. . .......... . ..

E9MLHLL01AIAXR RC ..A. .. .. C .. . .......... . ..

E9MLHLL01ALZF4 RC ..A. .. .. C .. . .......... . ..

E9MLHLL01ATP4N ..A. .. .. C .. . .......... . ..

E9MLHLL01AKEW3 ..A. .. .. C .. . .......... . ..

E9MLHLL01AGM41 ..A. .. .. C .. . .......... . ..

E9MLHLL01AH0X1 ..A. .. .. C .. . .......... . ..

E9MLHLL01ATDPL RC ..A. .. .. C .. . .......... . ..

E9MLHLL01APH2I ..A. .. .. C .. . .......... . ..

E9MLHLL01AGULS ..A. .. .. C .. . .......... . ..

E9MLHLL01AMZVO RC ..A. .. .. C .. . .......... . ..

E9MLHLL01AP7E8 RC ..A. .. .. C .. . .......... . ..

E9MLHLL01AGOGQ ..A. .. .. C .. . .......... . ..

E9MLHLL01AGT2S ..A. .. .. C .. . .......... . ..

E9MLHLL01ARZRL RC ..A. .. .. C .. . .......... . ..

E9MLHLL01AJGD6 ..A. .. .. C .. . .......... . ..

E9MLHLL01AIM91 ..A. .. .. C .. . .......... . ..

E9MLHLL01AK489 ..A. .. .. C .. . .......... . ..

E9MLHLL01ANXQQ ..A. .. .. C .. . .......... . ..

E9MLHLL01AJ23F ..A. .. .. C .. . .......... . ..

E9MLHLL01AQMQJ ..A. .. .. C .. . .......... . ..

E9MLHLL01ARLJ6 ..A. .. .. C .. . .......... . ..

E9MLHLL01AQPRA ..A. .. .. C .. . .......... . ..

E9MLHLL01ASHHQ ..A. .. .. C .. . .......... . ..

E9MLHLL01ARD54 RC ..A. .. .. C .. . .......... . ..

E9MLHLL01AQQOO ..A. .. .. C .. . .......... . ..

E9MLHLL01AJ3GN RC ..A. .. .. C .. . .......... . ..

E9MLHLL01AHO2E RC ..A. .. .. C .. . .......... . ..

E9MLHLL01AP1KX RC ..A. .. .. C .. . .......... . ..

E9MLHLL01AQZZM ..A. .. .. C .. . .......... . ..

E9MLHLL01AIZ9L ..A. .. .. C .. . .......... . ..

E9MLHLL01AOYEE ..A. .. .. C .. . .......... . ..

E9MLHLL01AM402 RC ..A. .. .. C .. . .......... . ..

E9MLHLL01AODWK ..A. .. .. C .. . .......... . ..

E9MLHLL01AK01Q RC ..A. .. .. C .. . .......... . ..

E9MLHLL01ATV38 ..A. .. .. C .. . .......... . ..

E9MLHLL01APIP1 ..A. .. .. C .. . .......... . ..

E9MLHLL01ARTIB RC ..A. .. .. C .. . .......... . ..

E9MLHLL01AGGXS RC ..A. .. .. C .. . .......... . ..

E9MLHLL01AINBC ..A. .. .. C .. . .......... . ..

E9MLHLL01AN03O ..A. .. .. C .. . .......... . ..

E9MLHLL01AHLB5 RC ..A. .. .. C .. . .......... . ..

E9MLHLL01AIN77 RC ..A. .. .. C .. . .......... . ..

E9MLHLL01AI9NV ..A. .. .. C .. . .......... . ..

E9MLHLL01AHRFN ..A. .. .. C .. . .......... . ..

E9MLHLL01ARJUV ..A. .. .. C .. . .......... . ..

E9MLHLL01AI24I ..A. .. .. C .. . .......... . ..

E9MLHLL01ARY7U RC ..A. .. .. C .. . .......... . ..

E9MLHLL01AQFA5 ..A. .. .. C .. . .......... . ..

E9MLHLL01ANOFA RC ..A. .. .. C .. . .......... . ..

E9MLHLL01AQJL0 RC ..A. .. .. C .. . .......... . ..

E9MLHLL01ANUZA ..A. .. .. C .. . .......... . ..

E9MLHLL01AQ7IT RC ..A. .. .. C .. . .......... . ..

E9MLHLL01AHGHZ ..A. .. .. C .. . .......... . ..

E9MLHLL01AGXNL RC ..A. .. .. C .. . .......... . ..

E9MLHLL01AJVF4 RC ..A. .. .. C .. . .......... . ..

E9MLHLL01AJS9Z ..A. .. .. C .. . .......... . ..

E9MLHLL01APSG3 RC ..A. .. .. C .. . .......... . ..

E9MLHLL01ANQPG ..A. .. .. C .. . .......... . ..

E9MLHLL01AJTI4 RC ..A. .. .. C .. . .......... . ..

E9MLHLL01ATFTA ..A. .. .. C .. . .......... . ..

E9MLHLL01AQHIW ..A. .. .. C .. . .......... . ..

E9MLHLL01ASILJ ..A. .. .. C .. . .......... . ..

E9MLHLL01ANUE9 ..A. .. .. C .. . .......... . ..

E9MLHLL01AIS5L RC ..A. .. .. C .. . .......... . ..

E9MLHLL01AHB0F ..A. .. .. C .. . .......... . ..

E9MLHLL01AMC5C RC ..A. .. .. C .. . .......... . ..

E9MLHLL01AIXYF RC ..A. .. .. C .. . .......... . ..

E9MLHLL01AQ5QM RC ..A. .. .. C .. . .......... . ..

E9MLHLL01APETW RC ..A. .. .. C .. . .......... . ..

E9MLHLL01ARH4G RC ..A. .. .. C .. . .......... . ..

E9MLHLL01ASBD1 RC ..A. .. .. C .. . .......... . ..

E9MLHLL01AKMX2 ..A. .. .. C .. . .......... . ..

E9MLHLL01AHAS4 ..A. .. .. C .. . .......... . ..

E9MLHLL01APL0V RC ..A. .. .. C .. . .......... . ..

E9MLHLL01APCBY ..A. .. .. C .. . .......... . ..

E9MLHLL01AJ4LN ..A. .. .. C .. . .......... . ..

E9MLHLL01AOXNE ..A. .. .. C .. . .......... . ..

E9MLHLL01AGY9J ..A. .. .. C .. . .......... . ..

E9MLHLL01ARPG3 ..A. .. .. C .. . .......... . ..

E9MLHLL01AO3TK ..A. .. .. C .. . .......... . ..

E9MLHLL01AKUIH ..A. .. .. C .. . .......... . ..

E9MLHLL01APRBX ..A. .. .. C .. . .......... . ..

E9MLHLL01AT8H7 ..A. .. .. C .. . .......... . ..

E9MLHLL01ANLGV RC ..A. .. .. C .. . .......... . ..

E9MLHLL01AG8PX ..A. .. .. C .. . .......... . ..

E9MLHLL01AIWL0 ..A. .. .. C .. . .......... . ..

E9MLHLL01AF1RW ..A. .. .. C .. . .......... . ..

E9MLHLL01AG6KB RC ..A. .. .. C .. . .......... . ..

E9MLHLL01AH31H RC ..A. .. .. C .. . .......... . ..

E9MLHLL01AIAPN RC ..A. .. .. C .. . .......... . ..

E9MLHLL01AHE4U RC ..A. .. .. C .. . .......... . ..

E9MLHLL01AJEJD RC ..A. .. .. C .. . .......... . ..

E9MLHLL01AL7LC RC ..A. .. .. C .. . .......... . ..

E9MLHLL01AGT70 RC ..A. .. .. C .. . .......... . ..

E9MLHLL01ASCL9 RC ..A. .. .. C .. . .......... . ..

E9MLHLL01AT5EP RC ..A. .. .. C .. . .......... . ..

E9MLHLL01AIVJ7 RC ..A. .. .. C .. . .......... . ..

E9MLHLL01AJUBT ..A. .. .. C .. . .......... . ..

E9MLHLL01AJDYG RC ..A. .. .. C .. . .......... . ..

E9MLHLL01AQX3L RC ..A. .. .. C .. . .......... . ..

E9MLHLL01ASUXX ..AA .. .. C .. . .......... . ..

E9MLHLL01ATI5U ..AA .. .. C .. . .......... . ..

E9MLHLL01AKBU2 RC ..A. .. .. C .. . .......A.. . ..

E9MLHLL01AHHUF RC ..AA .. .. C .. . .......... . ..

E9MLHLL01ATJFF RC ..AA .. .. C .. . .......... . ..

E9MLHLL01AKOVP RC ..A. .. .A C .. . .......... . ..

E9MLHLL01AQUXJ ..A. .. .. C A. . .......... . ..

E9MLHLL01AHL7C RC ..A. .. .A C .. . .......... . ..

E9MLHLL01AM4CG RC ..A. .. .. C .. . .......A.. . ..

E9MLHLL01AHPMW ..A. .. .. C A. . .......... . ..

E9MLHLL01ASTUH RC ..A. .. A. C .. . .......... . ..

E9MLHLL01AHVIP RC ..A. .. .A C .. . .......... . ..

E9MLHLL01AP2JK RC T.A. .. .. C .. . .......... . ..

E9MLHLL01ALMK3 ..A. .. .. C A. . .......... . ..

E9MLHLL01ANZJ6 RC ..A. .. .. C A. . .......... . ..

E9MLHLL01APN6Q ..A. .. .. C .. . ....T..... . ..

E9MLHLL01AJVVB RC ..A. .. .A C .. . .......... . ..

E9MLHLL01AJ75H ..A. .. A. C .. . .......... . ..

E9MLHLL01ANIES RC ..A. .. .A C .. . .......... . ..

E9MLHLL01ARIOY RC ..A. .. A. C .. . .......... . ..

E9MLHLL01AS0C5 RC A.A. .. .. C .. . .......... . ..

E9MLHLL01AOGAO RC ..A. .. .A C .. . .......... . ..

E9MLHLL01AK1QN ..A. T. .. C .. . .......... . ..

E9MLHLL01AKH8O RC ..A. .. .A C .. . .......... . ..

E9MLHLL01AR8HR RC ..A. .. A. C .. . .......... . ..

E9MLHLL01AG8V2 RC ..A. .. .. C .. . ....T..... . ..

E9MLHLL01AOWPA ..AA .. .. C .. . .......... . ..

E9MLHLL01AGTS1 RC ..A. .. .. C .T . .......... . ..

E9MLHLL01ASUPH RC ..A. .. .A C .. . .......... . ..

E9MLHLL01APKOZ RC ..A. .. .C C .. . .......... . ..

E9MLHLL01ASEDA ..A. .. .. C .T . .......... . ..

E9MLHLL01ATDA2 ..A. T. .. C .. . .......... . ..

E9MLHLL01AR5Q7 RC ..A. .. .. C A. . .......... . ..

E9MLHLL01AO91F ..A. .. .. C .. . ....T..... . ..

E9MLHLL01AJ3TA ..A. .. .. C .. . .......... A ..

E9MLHLL01AI98Y RC ..A. .. .. C .. .C.......... . ..

E9MLHLL01AKMIV ..A. .. .. C .. . .T........ . ..

E9MLHLL01AIR5Z RC ..A. .. .C C .. . .......... . ..

E9MLHLL01ATS9Z ..AA .. .. C .. . .......... . ..

E9MLHLL01AGJPM RC ..A. .. .. C ..T. .......... . ..

E9MLHLL01AGXEP RC ..A. .. .. C .T . .......... . ..

E9MLHLL01AI0QW RC ..A. . .. C .. . .......... . ..

E9MLHLL01AHUZF RC ..A. .. .. CC.. . .......... . ..

E9MLHLL01AKR7C ..A. .. .A C .. . .......... . ..

E9MLHLL01ALJYZ ..A. .. A. C .. . ......A... . ..

E9MLHLL01APUA4 RC ..A. .T .. T .. . .......... . ..

E9MLHLL01AOJXB RC ..A. .. A. C .. . ......A... . ..

E9MLHLL01AS0B8 ..A. .. A. C .. . ......A... . ..

E9MLHLL01AIBCB RC ..A. .. A. C .. . ......A... . ..

E9MLHLL01ARMFA RC ..A. .. A. C .. . ......A... . ..

E9MLHLL01AOWQD ..A. .. .. C .T . ......T... . ..

E9MLHLL01AS200 RC ..A. .. .. C .. .AC......... . ..

E9MLHLL01AM2WX ..A. .. .. C .. . AA........ . ..

E9MLHLL01AJYOZ RC ..A. .. A. C .. . ......A... . ..

E9MLHLL01AJ6ZX RC ..A. .. A. C .. . ......A... . ..

E9MLHLL01AFR82 RC ..A. .. .C C .. . . ........ . ..

E9MLHLL01AM88M RC ..A.A ..C.. C .. . ..........T. ..

E9MLHLL01AK4UG ..A.GA.. ..T.C.. . .......... .T..

Sidrón 1351c PCR1

EXON 6; 297; F20173 R20194

ABO2 11 TTGGCTGGCTCCCATTGTCTGGG AGGG CA TTC AACATCGACATCCTCAACGAGCAGTTCG

Consensus ABO2 11 . .... ..CA ... ........

E9MLHLL01AR7NE RC . .... ..CA ... ........

E9MLHLL01AN2XM RC . .... ..CA ... ........

E9MLHLL01AOGF5 . .... ..CA ... ........

E9MLHLL01AMH89 RC . .... ..CA ... ........

E9MLHLL01AKLB7 RC . .... ..CA ... ........

E9MLHLL01AG6R6 RC . .... ..CA ... ........

E9MLHLL01ALWBY . .... ..CA ... ........

E9MLHLL01AJFUM RC . .... ..CA ... ........

E9MLHLL01AIZ56 . .... ..CA ... ........

E9MLHLL01ARFZ4 RC . .... ..CA ... ........

E9MLHLL01AOAC8 RC . .... ..CA ... ........

E9MLHLL01ANWTC RC . .... ..CA ... ........

E9MLHLL01AJ4RU . .... ..CA ... ........

E9MLHLL01AIS5O . .... ..CA ... ........

E9MLHLL01AMOVW RC . .... ..CA ... ........

E9MLHLL01AIXI5 RC . .... ..CA ... ........

E9MLHLL01ANULS . .... ..CA ... ........

E9MLHLL01AQQEI RC . .... ..CA ... ........

E9MLHLL01AQD3E . .... ..CA ... ........

E9MLHLL01ARNAS RC . .... ..CA ... ........

E9MLHLL01AM1SQ RC . .... ..CA ... ........

E9MLHLL01ALQEB RC . .... ..CA ... ........

E9MLHLL01AHFK9 . .... ..CA ... ........

E9MLHLL01AQBNE . .... ..CA ... ........

E9MLHLL01AGPWM RC . .... ..CA ... ........

E9MLHLL01AQ4A6 RC . .... ..CA ... ........

E9MLHLL01AKSWO RC . .... ..CA ... ........

E9MLHLL01APF70 . .... ..CA ... ........

E9MLHLL01AIN89 . .... ..CA ... ........

E9MLHLL01AJFZ3 . .... ..CA ... ........

E9MLHLL01AKCNZ RC . .... ..CA ... ........

E9MLHLL01AOD4B RC . .... ..CA ... ........

E9MLHLL01AHGID RC . .... ..CA ... ........

E9MLHLL01AOIR2 . .... ..CA ... ........

E9MLHLL01AP5WJ RC . .... ..CA ... ........

E9MLHLL01ASOUG RC . .... ..CA ... ........

E9MLHLL01AM1S5 . .... ..CA ... ........

E9MLHLL01AI7IR RC . .... ..CA ... ........

E9MLHLL01AOG24 . .... ..CA ... ........

E9MLHLL01APF86 RC . .... ..CA ... ........

E9MLHLL01AGW6H RC . .... ..CA ... ........

E9MLHLL01ANWL4 RC . .... ..CA ... ........

E9MLHLL01AO9XO RC . .... ..CA ... ........

E9MLHLL01AKIZL RC . .... ..CA ... ........

E9MLHLL01AKE0L RC . .... ..CA ... ........

E9MLHLL01AGQJ8 . .... ..CA ...G........

E9MLHLL01ASXQC RC . .... ..CA ... ......C.

E9MLHLL01AOGNW RC . G... ..CA ... ........

E9MLHLL01AINQA .G....G..CA ... ........

E9MLHLL01ANTXV RC . .... ..ACCAC... ........

Sidrón 1351c PCR2

Mitochondrial control

mtDNA 6,267 TGCTCGCATCTGCTATAGTGGARG CC GGMGCA GGAACAGGTTGAACAGTCTACCCTCCCTTAGCA

Consensus CV4 13 ..A. .. ..C... .............

E9MLHLL01AR4L6 RC .... .. ...... .............

E9MLHLL01AK132 .... .. ...... .............

E9MLHLL01APPGH RC .... .. ...... .............

E9MLHLL01AH84N .... .. ...... .............

E9MLHLL01ASJ1N RC .... .. ...... .............

E9MLHLL01APHCZ .... .. ...... .............

E9MLHLL01AF10N .... .. ...... .............

E9MLHLL01AMLSU .... .. ...... .............

E9MLHLL01AFYXS .... .. ...... .............

E9MLHLL01AHLHM .... .. ...... .............

E9MLHLL01AG95N RC .... .. ...... .............

E9MLHLL01AIE10 .... .. ...... .............

E9MLHLL01AP086 .... .. ...... .............

E9MLHLL01APUXO .... .. ...... .............

E9MLHLL01ASHDB RC .... .. ...... .............

E9MLHLL01AK8FK .... .. ...... .............

E9MLHLL01AFSBE RC .... .. ...... .............

E9MLHLL01AIVB1 .... .. ...... .............

E9MLHLL01AL2TH .... .. ...... .............

E9MLHLL01AOS1N RC .... .. ...... .............

E9MLHLL01AIIS3 .... .. ...... .............

E9MLHLL01AMK18 .... .. ...... .............

E9MLHLL01AKEES RC .... .. ...... .............

E9MLHLL01AQTH9 .... .. ...... .............

E9MLHLL01AIQ4Y .... .. ...... .............

E9MLHLL01APQUX .... .. ...... .............

E9MLHLL01ANWF4 .... .. ...... .............

E9MLHLL01ASGSV RC ..A. .. ..C... .............

E9MLHLL01AL3FK ..A. .. ..C... .............

E9MLHLL01AR0YW ..A. .. ..C... .............

E9MLHLL01ANZJ9 ..A. .. ..C... .............

E9MLHLL01AG272 ..A. .. ..C... .............

E9MLHLL01AQPUM RC ..A. .. ..C... .............

E9MLHLL01AJ52T ..A. .. ..C... .............

E9MLHLL01AMJBG ..A. .. ..C... .............

E9MLHLL01AH07Z ..A. .. ..C... .............

E9MLHLL01AG2ZE RC ..A. .. ..C... .............

E9MLHLL01ALAYP ..A. .. ..C... .............

E9MLHLL01ALJU5 ..A. .. ..C... .............

E9MLHLL01AIUMM RC ..A. .. ..C... .............

E9MLHLL01AMYXW ..A. .. ..C... .............

E9MLHLL01AIXHQ ..A. .. ..C... .............

E9MLHLL01AGDBB ..A. .. ..C... .............

E9MLHLL01AN51M RC ..A. .. ..C... .............

E9MLHLL01AG5R4 RC ..A. .. ..C... .............

E9MLHLL01AJD2Q ..A. .. ..C... .............

E9MLHLL01AIKFX RC ..A. .. ..C... .............

E9MLHLL01ALK90 ..A. .. ..C... .............

E9MLHLL01ARX2S ..A. .. ..C... .............

E9MLHLL01AMU0Y ..A. .. ..C... .............

E9MLHLL01AR02Z ..A. .. ..C... .............

E9MLHLL01AI3E4 ..A. .. ..C... .............

E9MLHLL01AKR2Y ..A. .. ..C... .............

E9MLHLL01AS9OK ..A. .. ..C... .............

E9MLHLL01APE3F RC ..A. .. ..C... .............

E9MLHLL01AKB9X RC ..A. .. ..C... .............

E9MLHLL01AKD6J ..A. .. ..C... .............

E9MLHLL01AQP9K ..A. .. ..C... .............

E9MLHLL01AGWFJ RC ..A. .. ..C... .............

E9MLHLL01AR7B4 ..A. .. ..C... .............

E9MLHLL01AG17U ..A. .. ..C... .............

E9MLHLL01ANADH RC ..A. .. ..C... .............

E9MLHLL01AI2QM ..A. .. ..C... .............

E9MLHLL01AOFW0 RC ..A. .. ..C... .............

E9MLHLL01APH8A ..A. .. ..C... .............

E9MLHLL01AG2E8 ..A. .. ..C... .............

E9MLHLL01AOJ57 RC ..A. .. ..C... .............

E9MLHLL01AJDAT RC ..A. .. ..C... .............

E9MLHLL01AT15G ..A. .. ..C... .............

E9MLHLL01AI6XE ..A. .. ..C... .............

E9MLHLL01AF79G ..A. .. ..C... .............

E9MLHLL01ATV2F ..A. .. ..C... .............

E9MLHLL01AM8RW RC ..A. .. ..C... .............

E9MLHLL01AQ9N3 ..A. .. ..C... .............

E9MLHLL01ASOCS RC ..A. .. ..C... .............

E9MLHLL01AF33U RC ..A. .. ..C... .............

E9MLHLL01AN1PD RC ..A. .. ..C... .............

E9MLHLL01AQSSF RC ..A. .. ..C... .............

E9MLHLL01ANOSZ ..A. .. ..C... .............

E9MLHLL01ASRE5 ..A. .. ..C... .............

E9MLHLL01AP4WG ..A. .. ..C... .............

E9MLHLL01AJ95J ..A. .. ..C... .............

E9MLHLL01AM7BY RC ..A. .. ..C... .............

E9MLHLL01AQIEV ..A. .. ..C... .............

E9MLHLL01AL2T5 ..A. .. ..C... .............

E9MLHLL01ARVO7 RC ..A. .. ..C... .............

E9MLHLL01ALS7U RC ..A. .. ..C... .............

E9MLHLL01AG5XQ RC ..A. .. ..C... .............

E9MLHLL01AQML9 ..A. .. ..C... .............

E9MLHLL01ATMZD RC ..A. .. ..C... .............

E9MLHLL01AQDY7 RC ..A. .. ..C... .............

E9MLHLL01APYWL ..A. .. ..C... .............

E9MLHLL01AI617 ..A. .. ..C... .............

E9MLHLL01AQS37 RC ..A. .. ..C... .............

E9MLHLL01AR2CD ..A. .. ..C... .............

E9MLHLL01AHRVQ RC ..A. .. ..C... .............

E9MLHLL01ASWY0 RC ..A. .. ..C... .............

E9MLHLL01AMXR8 RC ..A. .. ..C... .............

E9MLHLL01AMZNJ ..A. .. ..C... .............

E9MLHLL01AKZ6A RC ..A. .. ..C... .............

E9MLHLL01ATM3M ..A. .. ..C... .............

E9MLHLL01AIROM ..A. .. ..C... .............

E9MLHLL01AJMIQ ..A. .. ..C... .............

E9MLHLL01AQBI1 RC ..A. .. ..C... .............

E9MLHLL01AI9DB ..A. .. ..C... .............

E9MLHLL01AN0FR RC ..A. .. ..C... .............

E9MLHLL01AP7JB RC ..A. .. ..C... .............

E9MLHLL01AQ2P2 ..A. .. ..C... .............

E9MLHLL01ARJTP ..A. .. ..C... .............

E9MLHLL01AN7K6 RC ..A. .. ..C... .............

E9MLHLL01AR8PR ..A. .. ..C... .............

E9MLHLL01AN7T7 RC ..A. .. ..C... .............

E9MLHLL01AQQ63 RC ..A. .. ..C... .............

E9MLHLL01AKCR7 RC ..A. .. ..C... .............

E9MLHLL01AFYNS ..A. .. ..C... .............

E9MLHLL01AP1Y4 ..A. .. ..C... .............

E9MLHLL01AKSJE ..A. .. ..C... .............

E9MLHLL01ALXQ0 ..A. .. ..C... .............

E9MLHLL01AIEHX ..A. .. ..C... .............

E9MLHLL01AIU52 ..A. .. ..C... .............

E9MLHLL01AHOGL RC ..A. .. ..C... .............

E9MLHLL01APR61 RC ..A. .. ..C... .............

E9MLHLL01ASB62 ..A. .. ..C... .............

E9MLHLL01ANLCY ..A. .. ..C... .............

E9MLHLL01ATC52 ..A. .. ..C... .............

E9MLHLL01AGE0D ..A. .. ..C... .............

E9MLHLL01ARS58 RC ..A. .. ..C... .............

E9MLHLL01AIH6D ..A. .. ..C... .............

E9MLHLL01ATCHD RC ..A. .. ..C... .............

E9MLHLL01AF45L RC ..A. .. ..C... .............

E9MLHLL01ARNIF ..A. .. ..C... .............

E9MLHLL01AR2KM RC ..A. .. ..C... .............

E9MLHLL01AQEL1 ..A. .. ..C... .............

E9MLHLL01ALRFQ RC ..A. .. ..C... .............

E9MLHLL01AKGJL ..A. .. ..C... .............

E9MLHLL01AI1X1 RC ..A. .. ..C... .............

E9MLHLL01AG3H3 ..A. .. ..C... .............

E9MLHLL01AIDMU ..A. .. ..C... .............

E9MLHLL01ATPIZ ..A. .. ..C... .............

E9MLHLL01AHJSB ..A. .. ..C... .............

E9MLHLL01AK5N1 ..A. .. ..C... .............

E9MLHLL01AHAG8 RC ..A. .. ..C... .............

E9MLHLL01AF48M RC ..A. .. ..C... .............

E9MLHLL01AGMN9 RC ..A. .. ..C... .............

E9MLHLL01AHJ1B RC ..A. .. ..C... .............

E9MLHLL01AGHTY RC ..A. .. ..C... .............

E9MLHLL01AGXW0 RC ..A. .. ..C... .............

E9MLHLL01APOUE RC ..A. .. ..C... .............

E9MLHLL01AGHYE RC ..A. .. ..C... .............

E9MLHLL01AQXXW RC ..A. .. ..C... .............

E9MLHLL01AIQT4 RC ..A. .. ..C... .............

E9MLHLL01AO3X5 RC ..A. .. ..C... .............

E9MLHLL01AMJ2P RC ..A. .. ..C... .............

E9MLHLL01AOF0E ..A. .. ..C... .............

E9MLHLL01APPHZ RC ..A. .. ..C... .............

E9MLHLL01APFN2 RC ..A. .. ..C... .............

E9MLHLL01AI6IG RC ..A. .. ..C... .............

E9MLHLL01ARC53 ..A. .. ..C... .............

E9MLHLL01AH8LL ..A. .. ..C... .............

E9MLHLL01AH7F7 ..A. .. ..C... .............

E9MLHLL01ATSOO ..A. .. ..C... .............

E9MLHLL01AN6Q9 RC ..A. .. ..C... .............

E9MLHLL01AKL7W ..A. .. ..C... .............

E9MLHLL01AIVMJ RC ..A. .. ..C... .............

E9MLHLL01AHXX2 ..AA .. ..C... .............

E9MLHLL01AINEQ ..A. .. ..C... .A...........

E9MLHLL01APM4Z ..A. .. ..C... . ...........

E9MLHLL01AF45B ..A. .. ..CA.. .............

E9MLHLL01ASK10 RC ..A. T. ..C... .............

E9MLHLL01AIOKS RC ..A. .. ..C... ......T......

E9MLHLL01ATL2Z ..A. T. ..C... .............

E9MLHLL01AJFP9 RC ..A. .. ..C...C.............

E9MLHLL01AOU9R ..A. T. ..C... .............

E9MLHLL01AHME5 .GA. .. ..C... .............

E9MLHLL01AP2AM ..A. T. ..C... .............

E9MLHLL01AFX2F ..A .. ..C... .............

E9MLHLL01AS301 RC A.A. .. ..C... .............

E9MLHLL01AKVMV ..A. .. ..C... ..G..........

E9MLHLL01AMQCJ ..A. T. ..C... .............

E9MLHLL01AMB1J ..A. .. ..C... .............

E9MLHLL01ARM0E ..A. T. ..C... .............

E9MLHLL01AF09L RC ..A. .. ..C... .............

E9MLHLL01AP3VH RC ..A. .. ..C... ....T........

E9MLHLL01AMZEO RC ..A. .. ..C... ....T........

E9MLHLL01AR89M RC ..A. T. ..C... .............

E9MLHLL01ARRMQ RC ..A. ..C...... .............

E9MLHLL01AJ4LE RC ..A. TT ..C... .............

E9MLHLL01AGJ0P RC ..A. TT ..C... .............

E9MLHLL01AF7RZ RC ..A. TT ..C... .............

E9MLHLL01AREYY RC ..A. TT ..C... .............

E9MLHLL01ALGQ1 RC ..A. TT ..C... .............

E9MLHLL01ANOOF RC ..A. TT ..C... .............

E9MLHLL01AN6EJ RC ..A. TT ..C... .............

E9MLHLL01APRKT ..A. .. ..C... A...........

E9MLHLL01AP41O ..A. .. .AC... A............

E9MLHLL01ARJOK RC ..A. TT ..C... .............

E9MLHLL01APL84 RC ..A. TT ..C... .............

E9MLHLL01AK2JX ..AT . ..C... .............

E9MLHLL01AHJO6 RC ..A. .. ..C... AA...........

E9MLHLL01AN86D RC ..A. TT ..C... .............

E9MLHLL01AH4AO RC ..A. .. ..C... ......AA.....

E9MLHLL01AO5H1 RC ..A. .T ..C.T. .............

E9MLHLL01AS2Z5 RC ..A. TT ..C... .............

E9MLHLL01AJW8E RC ..A. TT ..C... .............

E9MLHLL01AKKRS RC ..A. .T ..C.T. ....T........

E9MLHLL01AP4MH RC ..A. .T ..C.T. ....T........

E9MLHLL01AF7HG RC ..A.G.. ..CN.. A...........

E9MLHLL01AKGC3 RC ..A. .T ..C.T. ....T........

E9MLHLL01AG037 RC ..A. .T ..C.T. ....T........

E9MLHLL01ASOV8 ..A. .T ..C.T. ....T........

E9MLHLL01AQFK2 ..A. TT ..T.T. .............

E9MLHLL01AR1SO RC ..A. TT ..T.T. .............

E9MLHLL01AP4A5 ..A. TT ..T.T. .............

Sidron 1351c PCR2

EXON 6; 261; F20144 R20163

ABO1 10 AGGAAGGATGTCCTCGTGGT ACCCC TT GG C TGGC TCCCATTGTCTGGGAGGGCA

Consensus ABO1 10 .G ..... .. .. . .... ..

E9MLHLL01ARO51 RC .G ..... .. .. . .... ..

E9MLHLL01AKEJU RC .G ..... .. .. . .... ..

E9MLHLL01ASFOG RC .G ..... .. .. . .... ..

E9MLHLL01AJNM0 RC .G ..... .. .. . .... ..

E9MLHLL01AL95V RC .G ..... .. .. . .... ..

E9MLHLL01ASANN RC .G ..... .. .. . .... ..

E9MLHLL01AIFDD .G ..... .. .. . .... ..

E9MLHLL01APKTI .G ..... .. .. . .... ..

E9MLHLL01AMPY1 RC .G ..... .. .. . .... ..

E9MLHLL01AN3AL RC .G ..... .. .. . .... ..

E9MLHLL01AIAFF .G ..... .. .. . .... ..

E9MLHLL01AL3UD RC .G ..... .. .. . .... ..

E9MLHLL01ARXW4 .G .... .. .. . .... ..

E9MLHLL01AGSQD .G .....C..C..G. .... ..

E9MLHLL01AMOIY .G .... .. .. . .... ..

E9MLHLL01AH8J7 RC .G ..... .. .. . .... ..

E9MLHLL01AIE22 .G .... .. .. . .... ..

E9MLHLL01AK110 RC .G ..... .. .. . .... ..

E9MLHLL01AQLD4 RC .G ..... .. .. . .... ..

E9MLHLL01ATGE6 RC .G ..... .. .. . .... ..

E9MLHLL01ANLIW RC .G ..... .. .. .C....C..

E9MLHLL01APIN4 .G .... .. .. . .... ..

E9MLHLL01AI98V RC .G ..... .. A. . .... ..

E9MLHLL01AS634 .G .... .. .. . .... ..

E9MLHLL01ANN8A .G .... .. .. . .... ..

E9MLHLL01AS34U .G .... .. .. . .... ..

E9MLHLL01ARO5Y RC .G ..... .. .. . .... .

E9MLHLL01ANRQ5 RC .G ..... .. .. . .... ..

E9MLHLL01ATF2E .G ..... .. .. . .... ..

E9MLHLL01ANBWH .G ..... .. .. . .... ..

E9MLHLL01ASLGJ RC .G ..... .. .. . .... N.

E9MLHLL01ALK53 RC .G ..... .. .. . .... ..

E9MLHLL01AJLTE RC .G ..... .. .. . .... ..

E9MLHLL01ANNZ9 .G ..... .. .. . .... ..

E9MLHLL01AR7VU .G ..... .. .. . .... ..

E9MLHLL01AN4PL .G ..... .. .. . .... ..

E9MLHLL01AS065 RC .G ..... .. .. . .... ..

E9MLHLL01AHJRD .G ..... .. .. . .... ..

E9MLHLL01ASZVB RC .G ..... .. .. . .... ..

E9MLHLL01AQECD .G ..... .. .. . .... ..

E9MLHLL01AG350 RC .G ..... .. .. . .... ..

E9MLHLL01AGTH0 RC .G ..... .. .. . .... ..

E9MLHLL01AIHFJ .G ..... .. .. . .... ..

E9MLHLL01AQWHP RC . ..... .. .. . .... ..

E9MLHLL01AOSMH . ..... .. .. . .... ..

E9MLHLL01AN7HH . ..... .. .. . .... ..

E9MLHLL01ARH3B RC . ..... .. .. . .... ..

E9MLHLL01AO89T RC . ..... .. .. . .... ..

E9MLHLL01ARSXP RC . ..... .. .. . .... ..

E9MLHLL01ASEWH RC . ..... .. .. . .... ..

E9MLHLL01AJ240 RC . ..... .. .. . .... ..

E9MLHLL01AHI1T RC . ..... .. .. . .... ..

E9MLHLL01AQDNF . ..... .. .. . .... ..

E9MLHLL01ANHT3 RC . ..... .. .. . .... ..

E9MLHLL01AGWO5 RC . ..... .. .. . .... ..

E9MLHLL01ALAX2 . ..... .. .. . .... ..

E9MLHLL01AHFWN RC . ..... .. .. . .... ..

E9MLHLL01AKIZY RC . ..... .. .. . .... ..

E9MLHLL01AMANY . ..... .. .. . .... ..

E9MLHLL01ANUJ5 RC . ..... .. .. . .... ..

E9MLHLL01AIU0B RC . ..... .. .. . .... ..

E9MLHLL01ASNZX RC . ..... .. .. . .... ..

E9MLHLL01ANAZO . ..... .. .. . .... ..

E9MLHLL01AONYB . ..... .. .. . .... ..

E9MLHLL01AI6I7 RC . ..... .. .. . .... ..

E9MLHLL01AI53B . ..... .. .. . .... ..

E9MLHLL01AJKHY . .... .. .. . .... ..

E9MLHLL01ATJVM . .... .. .. . .... ..

E9MLHLL01AG813 . .... .. .. . .... ..

E9MLHLL01AN748 . .... .. .. . .... ..

E9MLHLL01AQQTA RC . .... .. .. . .... ..

E9MLHLL01ARY1G . .... .. .. . .... ..

E9MLHLL01ATSFX RC . ..... .. .. . .... ..

E9MLHLL01ANWN1 . .... .. .. . .... ..

E9MLHLL01AP5E8 . .... .. .. . .... ..

E9MLHLL01AJ463 . .... .. .. . .... ..

E9MLHLL01AHMDS . .... .. .. . .... ..

E9MLHLL01AFUY1 RC . ..... .. .. . ....C..

E9MLHLL01ARQK9 . .... .. .. . .... ..

E9MLHLL01AHGAH . .... .. .. . .... ..

E9MLHLL01AMRFH . .... .. .. . .... ..

E9MLHLL01AIAEQ . .... .. .. . .... ..

E9MLHLL01ASOBR .... .. . . .... ..

E9MLHLL01ATSSS .... . . . .. . ..

Sidrón 1351c PCR2

EXON 6; 297; F20173 R20194

ABO2 11 TTGGCTGGCTCCCATTGTCTGGG AGGG CA TTCAAC ATCGACATCCTCAACGAGCAGTTC

Consensus ABO2 11 . .... ..CA...... .....

E9MLHLL01ATVRX RC . .... ..CA...... .....

E9MLHLL01AOARQ RC . .... ..CA...... .....

E9MLHLL01AIRCB . .... ..CA...... .....

E9MLHLL01ASEPH RC . .... ..CA...... .....

E9MLHLL01AS6JP RC . .... ..CA...... .....

E9MLHLL01AIWML RC . .... ..CA...... .....

E9MLHLL01APU77 RC . .... ..CA...... .....

E9MLHLL01ASWM2 RC . .... ..CA...... .....

E9MLHLL01AS3PG RC . .... ..CA...... .....

E9MLHLL01AI6W6 . .... ..CA...... .....

E9MLHLL01AKFKO RC . .... ..CA...... .....

E9MLHLL01AG90M . .... ..CA...... .....

E9MLHLL01AGJ53 . .... ..CA...... .....

E9MLHLL01ALTLU . .... ..CA...... .....

E9MLHLL01AQPRJ RC . .... ..CA...... .....

E9MLHLL01AL6Y5 . .... ..CA...... .....

E9MLHLL01ATS8U . .... ..CA...... .....

E9MLHLL01AGRQH . .... ..CA...... .....

E9MLHLL01APLPQ . .... ..CA...... .....

E9MLHLL01AP2QA RC . .... ..CA...... .....

E9MLHLL01AQUQP . .... ..CA...... .....

E9MLHLL01AFYLQ RC . .... ..CA...... .....

E9MLHLL01AOMJY RC . .... ..CA...... .....

E9MLHLL01ALWBP RC . .... ..CA...... .....

E9MLHLL01ALADX RC . .... ..CA...... .....

E9MLHLL01AN3PF RC . .... ..CA...... .....

E9MLHLL01ARMEW RC . .... ..CA...... .....

E9MLHLL01AS6B2 RC . .... ..CA...... .....

E9MLHLL01AQBTZ . .... ..CA...... .....

E9MLHLL01ANWXK . .... ..CA...... .....

E9MLHLL01AGOA9 . .... ..CA...... .....

E9MLHLL01AOF2H RC . .... ..CA...... .....

E9MLHLL01AJR70 RC . .... ..CA...... .....

E9MLHLL01ALTG3 . .... ..CA...... .....

E9MLHLL01AQEWM . .... ..CA...... .....

E9MLHLL01AOFFJ RC . .... ..CA...... .....

E9MLHLL01AF14X RC . .... ..CA...... .....

E9MLHLL01AQM9Z . .... ..CA...... .....

E9MLHLL01ATVKI . .... ..CA...... .....

E9MLHLL01AOG28 RC . .... ..CA...... .....

E9MLHLL01ALSKZ . .... ..CA......C.....
